# Indigo Beyond Tradition: Scalable Derivatization, Extraction from Waste Denim Textiles, and Boc-Protected Intermediates

**DOI:** 10.1021/acs.joc.5c02380

**Published:** 2025-12-31

**Authors:** Gökhan Kaplan, Nurgül Seferoğlu, Ertan Şahin, Zeynel Seferoğlu

**Affiliations:** † Department of Chemistry, Graduate School of Natural and Applied Sciences, Gazi University, Yenimahalle, 06560 Ankara, Türkiye; ‡ 473499 Sanko Tekstil İşletmeleri Sanayi ve Ticaret A.S. İsko Sb, Organize Sanayi Bölgesi 3. Cadde, 16400 İnegöl, Bursa, Türkiye; § Department of Advanced Technology, Graduate School of Natural and Applied Sciences, Gazi University, Yenimahalle, 06560 Ankara, Türkiye; ∥ Department of Chemistry, Faculty of Science, 37503 Atatürk University, 25240 Erzurum, Türkiye; ⊥ Department of Chemistry, Faculty of Science, 175163 Gazi University, Yenimahalle, 06560 Ankara, Türkiye

## Abstract

This study demonstrates the feasibility of industrial-scale, column-free synthesis of *tert*-butoxycarbonyl (Boc)-protected indigo derivatives and highlights their application potential in the textile industry for the dyeing of previously undyeable synthetic textiles with indigo. The direct synthesis of *N*-mono-*tert*-butoxycarbonyl (Boc) indigo, an important intermediate for indigo substitution studies, was achieved under very mild conditions and in a moderate yield. For the first time, the molecular structures of all synthesized compounds were comprehensively characterized through detailed NMR and HRMS analyses as well as the single-crystal X-ray diffraction method. The influence of the Boc groups on the photophysical and thermal properties, oxidation–reduction behavior, and dyeing performance was thoroughly investigated and defined. Importantly, the oxidation mechanism of dihydro-di-Boc indigo (i.e., reduced di-Boc indigo) was elucidated by combined experimental and theoretical approaches. Furthermore, this study shows that indigo-dyed textile waste can serve as valuable starting materials for the synthesis of Boc-protected indigo derivatives. This innovative methodology offers significant promise not only for textile-to-textile recycling technologies but also as a sustainable strategy to address global waste management and environmental pollution challenges.

## Graphical abstract

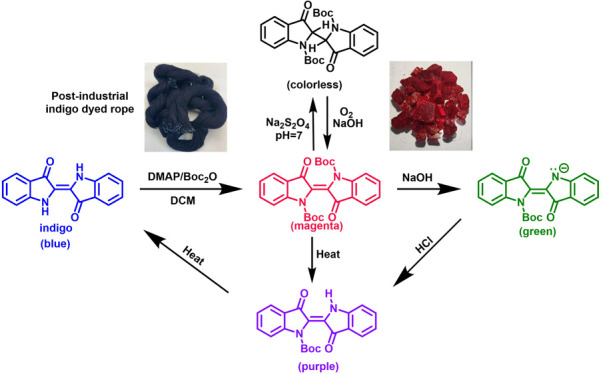

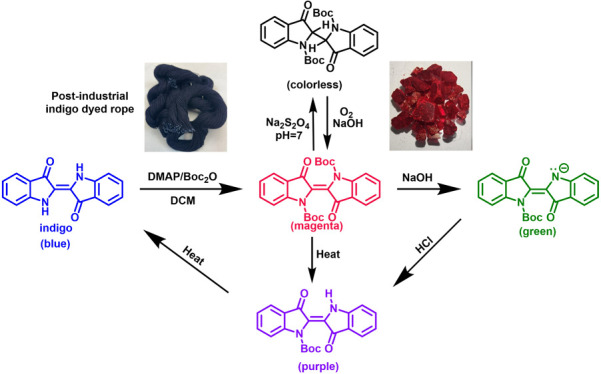

## Introduction

The coloration of materials (textiles, glass, walls, plastics, vehicles, furniture, body parts, etc.) remains a subject of significant interest not only within the fashion industry but also in scientific research. To date, over a hundred classes of dyes have been studied and utilized across a wide range of applications. Indigo is one of these dyes that has been known for over 6000 years^1^ and has been used in dyeing throughout history as Nature’s gift to humanity. Scientific studies on indigo and its derivatives have increased significantly following its successful synthesis and structural characterization. As of 2023, the annual production of indigo exceeds 80,000 tons, with the majority being utilized in the manufacturing of denim.^2^ Denim is typically produced from cotton or cotton–polyester–elastane blend fibers, which are dyed with indigo using a conventional vat dyeing process that involves reduction and oxidation steps to overcome the problems related to indigo’s insolubility in water.^3^ In addition to its use in denim production, indigo and its derivatives have been applied across a diverse range of fields, including organic electronics,^4^ photoswitches,^4b,5^ functional textiles (as a chromophore),^6^ medical, cosmetics and food applications,^7^ battery technology,^8^ photovoltaics,^9^ energy storage and biological systems.^10^ Due to remarkable characteristics, both identified and yet to be revealed, of indigo and its derivatives, the interest in its functionalization through carbonyl, amine, and benzene rings (Figure 1) continues to grow annually. Using aromatic amines, Nindigo compounds are synthesized through carbonyl substitution,^11^ and Grignard reagents are employed to produce nonsymmetrical diindoles.^12^ Bis-indigotin molecule represents another example of carbonyl-functionalized indigo, which is naturally designed and synthesized in nature.^13^ Although numerous scientific studies have examined ring-substituted indigo derivatives at the ortho (4,4′) and (7,7′) positions, as well as the para (5,5′) and (6,6′) positions, most of these compounds have been synthesized from precursors other than indigo itself, such as substituted 2-nitrobenzaldehyde or 2-bromobenzoic acid.^3a,4c^ The predominant focus of research revolves around the *N*-substitution of indigo, encompassing various modification strategies such as alkylation,^4b,14^ allylation,^15^ acylation,^4b,10,14a,16^ annulation,^17^ and arylation.^4b,10,18^

**Figure 1:**
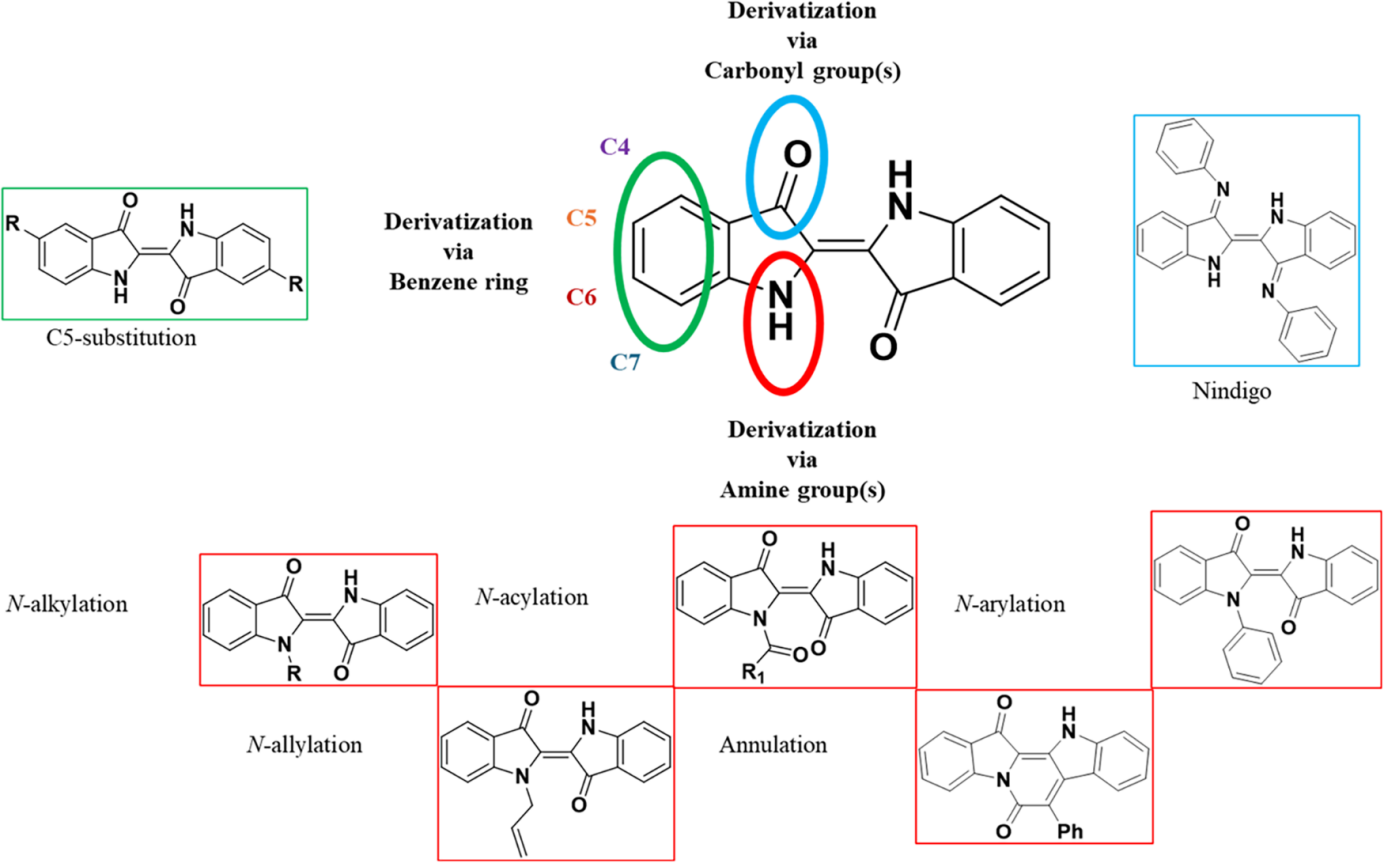
Derivatization of indigo through its functional groups.

Although various approaches have been proposed for the synthesis of indigo derivatives to date, several parameters still require improvement, especially for large-scale applicability. These include harsh reaction conditions, the need for catalyst(s), multiple synthesis steps, low yields, the high solvent consumption requirement due to the low solubility of indigo, and challenges in isolation and purification methods. In addition to these challenges, the pollution potential and overall environmental impact of synthetic processes must be thoroughly assessed to ensure that the processes are not only efficient but also sustainable and environmentally friendly.

In this study, we report a new synthesis and isolation approach for *N*,*N*′-di­(Boc)­indigo (**1**), a new synthetic method for *N*-mono­(Boc)­indigo (**2**), and a novel method for *N*,*N*′-di­(Boc)-dihydroindigo (**3**) starting from indigo compound extracted from waste denim textiles, including postconsumer, preconsumer, and postindustrial feedstocks (Figure 2A). Dye **1** is also synthesized on a kilogram scale, implementing washing steps in the isolation procedure (column-free isolation/purification). The structural and thermal characterization of the synthesized compounds was obtained using NMR, FTIR, HRMS, and TGA, with single-crystal X-ray analysis providing detailed confirmation of the molecular structures. Furthermore, the photophysical properties of all compounds were investigated through UV/vis and fluorescence spectroscopy. The potential tautomeric properties of **3** were evaluated experimentally and theoretically, and the most stable tautomeric structure was determined. The proposed oxidation mechanism of **3** was further elucidated by a combination of ^1^H NMR titration, experimental studies, and theoretical analyses. Finally, **1** was applied in textile dyeing processes to dye synthetic materials (polyethylene terephthalate (**PET**), polypropylene (**PP**), and polyurethane/Elastane (**PU**)) that cannot be colored using the conventional indigo dyeing methods.

**Figure 2:**
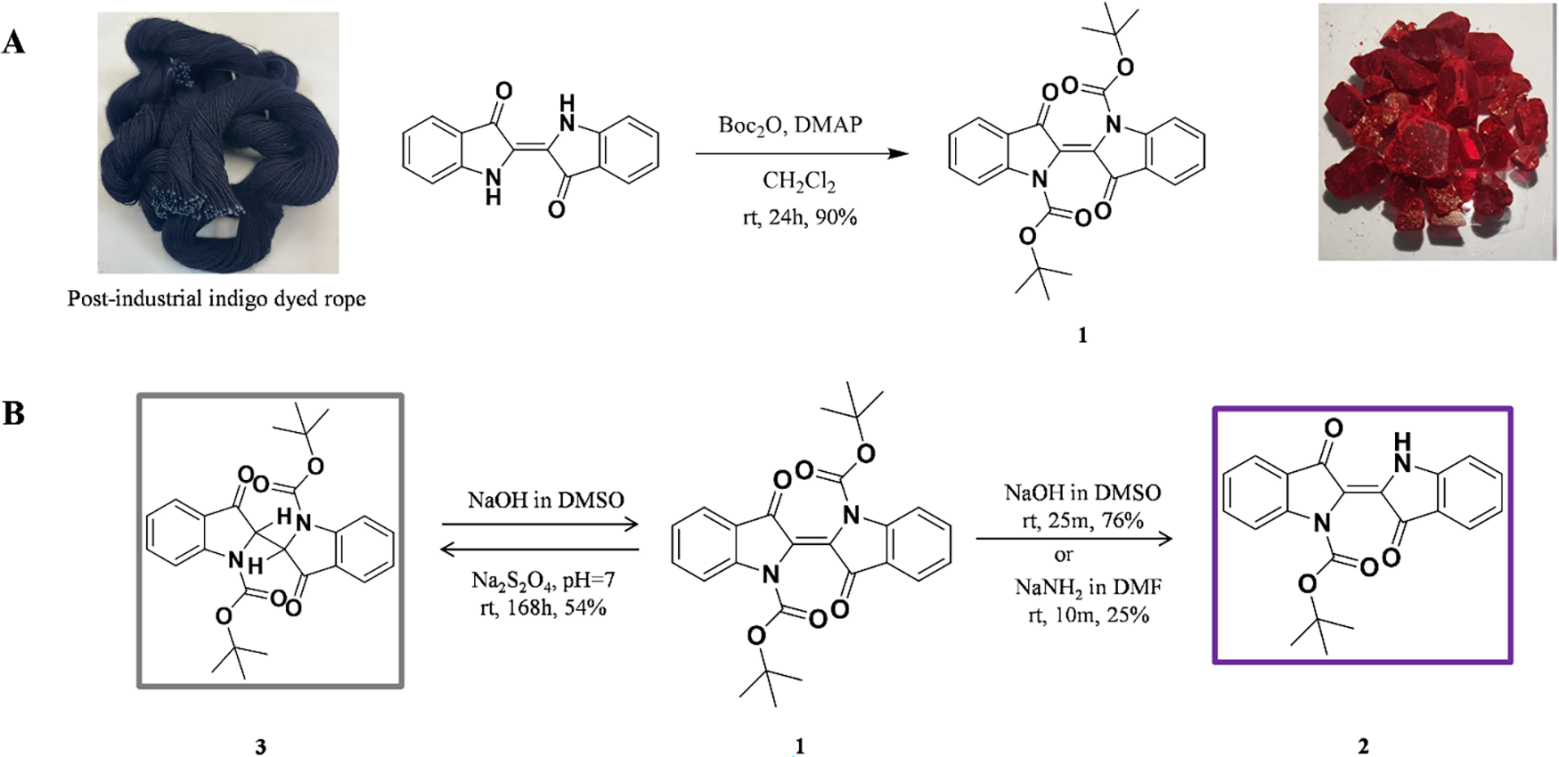
Novel synthesis methodology for *N*-substituted indigo (A) synthesis of **1** from waste feedstocks; (B) synthesis of **2** and **3** from **1**.

## Results and Discussion

### Synthesis and Characterization

The synthesis of dye **1** on a kilogram scale was carried out following the procedure reported by Sarıçiftçi et al. in 2013,^16a^ utilizing a fully explosion-proof reactor system (Figure S1 in the Supporting Information). As column chromatography was not feasible and environmentally friendly for the isolation and purification of **1** on an industrial scale (due to the requirement of a large amount of solvent(s) and silica gel consumption), a series of solvent-free washing steps, including alkaline, acidic, and neutral water washes, were developed and implemented. Furthermore, to enhance economic viability and reduce the environmental impact of the process, the solvent used in the synthesis was recovered through evaporation and reused in several batches of **1** synthesis. Due to the reaction mechanism, *tert*-butyl alcohol is an inherently present in reaction medium and remains in the solvent even after evaporation; as a result, the reaction yield started to decrease after the fourth cycle, which correlates with its accumulated concentration (Figure S3 in the Supporting Information). Dye **1** was also synthesized using a new approach starting from indigo existing in textile waste dyed with indigo, including postindustrial, preconsumer, and postconsumer feedstocks, obtaining a moderate yield (50–55%). This method offers several key advantages: (i) the valorization of textile waste through the synthesis of high-value materials, (ii) a reduction in the environmental impact associated with conventional indigo production processes, and (iii) the mitigation of one of the biggest challenges in textile recycling technologies, dye stripping from waste fabrics.^19^

In 2021, Melo et al. synthesized both **1** and **2** as a mixture using microwave-assisted methods and obtained them in almost equal low yields of 33 and 30%, respectively, which necessitated their individual isolation by column chromatography. In contrast to this approach, we succeeded in the direct synthesis of **2** from **1** in the presence of NaOH in DMSO or NaNH_2_ in DMF within a few minutes at room temperature, with good and low yields of 76% in DMSO and 25% in DMF, respectively (Figure 2B). The molecular structure of **2** was elucidated for the first time through a single-crystal X-ray diffraction analysis. Additionally, comprehensive characterization was carried out using NMR, FTIR, and HRMS techniques. A pronounced N–H signal at about 10 ppm in CDCl_3_ is indicative of **2**, and the loss of symmetry in **2** results in ten individual proton resonances in its NMR spectrum (Figure S17 in the Supporting Information). The significance of **2** lies in its role as a precursor for indigo functionalization reactions via the nitrogen atom. Both symmetric and asymmetric (aliphatic and aromatic) substitutions can be carried out under mild conditions, enabling (i) functionalization via one nitrogen atom, (ii) removing of the Boc group, and (iii) subsequent functionalization via the deprotected nitrogen atom.

Indigo must be reduced prior to the dyeing process due to its insolubility in water.^3a,3b^ The reduced, solubilized form of indigo can exist in different ionic states like nonionic, monoionic, and di-ionic depending on the pH of the environment^3b^ (Figure 3). The nonionic form, commonly referred to in literature as acidic leuco-indigo, prereduced indigo, or indigo-white, has been the subject of various patents and scientific papers concerning its synthesis and stabilization due to its sensitivity to oxygen.^3b,20^ Here, we synthesized **3**, a reduced form of indigo, directly from **1** as a light gray solid in the presence of sodium dithionite (Na_2_S_2_O_4_) under neutral pH conditions at room temperature with a moderate yield of 54% as the keto form of acidic leuco-indigo. (Figure 2B). In contrast to previously reported studies,^3b,20^ we showed that the keto form of **3** is a more stable tautomer, which is insoluble in water and exhibits greater stability against atmospheric oxygen. DFT calculations also revealed that the diketo form is more stable in the gas phase and in solvents than the enol and di-enol forms forms (Figure 4). The molecular structure of **3** was confirmed by single-crystal X-ray diffraction analysis. In addition, a comprehensive characterization was conducted using NMR spectroscopy, FTIR, and HRMS (Figures S14, S15, and S18 in the Supporting Information). A clear signal at around 4.7 ppm is indicative of **3**, while five other well-resolved signals appear in both the aromatic and aliphatic regions of the NMR spectrum, consistent with the retained molecular symmetry after reduction.

**Figure 3:**
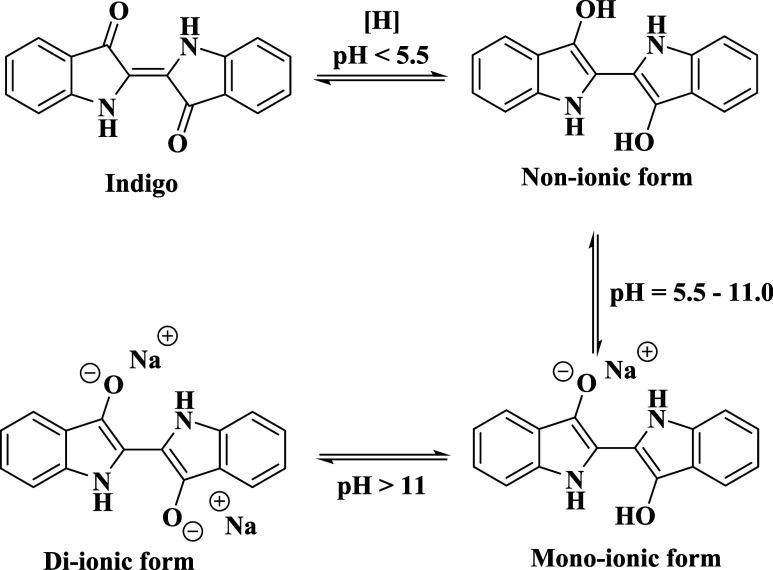
Molecular structures of reduced indigo at different pH.

**Figure 4:**
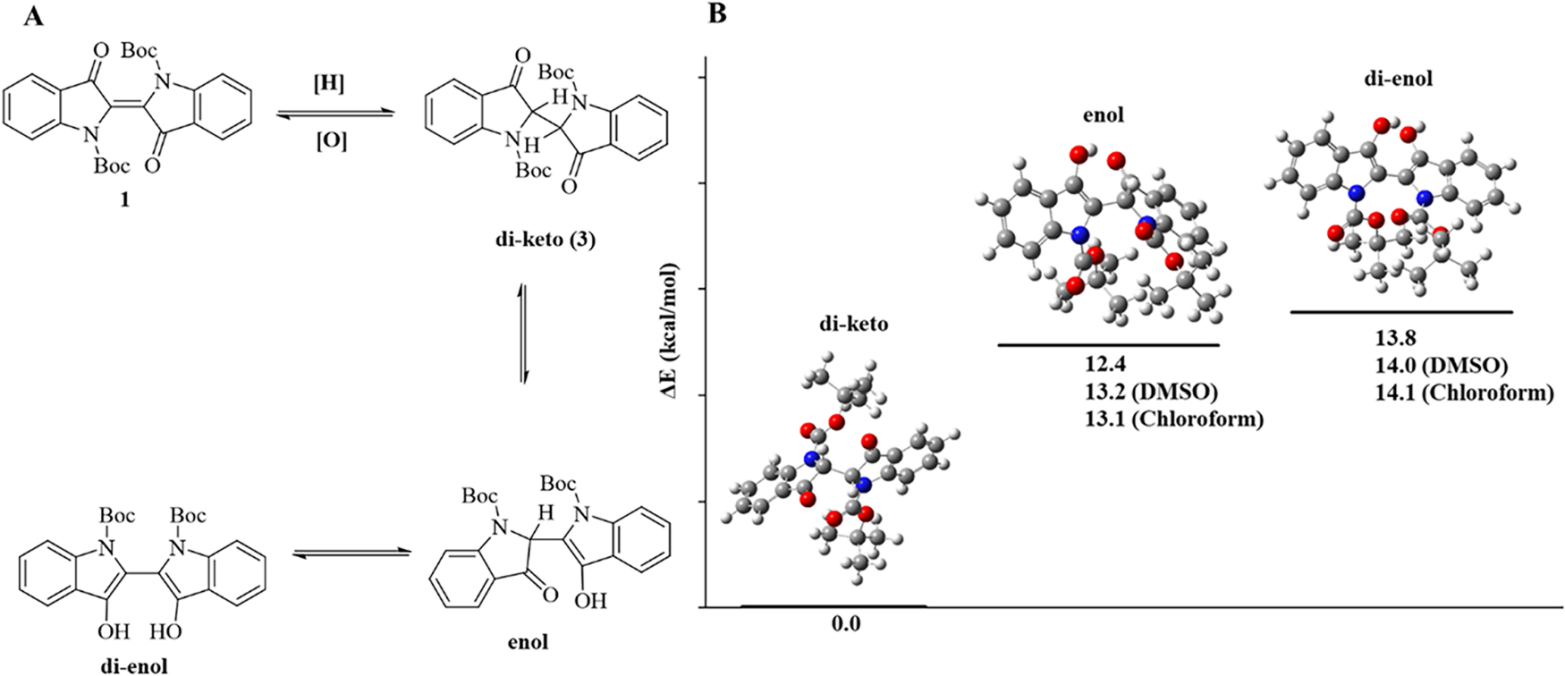
Possible tautomers of **3** (A) and stability of tautomeric forms of **3** (B) in the gas phase and solvents at the B3LYP/6–311+G­(d,p) level.

Single-crystal X-ray structure analyses of **2** and **3** were performed. **2** is crystallized in the monoclinic P21/n space group, and **3** is crystallized in the orthorhombic Pcba space group. Again, from X-ray analysis, we know that the indigo B molecule is almost planar and in *E*-conformation.^21^ In **2**, the Boc group is only bonded to one of the indolin-3-one groups via the nitrogen atom. Here, the indigo group deviates from planarity. The C13=C14 [1.344(3) Å] bond is shorter than the C=C double bond in the indigo B molecule [1.359(3) Å].

In the indolin-3-one group to which Boc is attached, the C–N1 bond lengths are longer [1.400(3)–1.425(3) Å] than C–N2 [1.357(3)–1.383(3) Å]. In the indigo B structure, the C–N bond length in the indolin-3-one varies between 1.382(3) and 1.394(3) Å. The carbonyl C=O [1.211(3)–1.218(3) Å] double bond in **2** is much shorter than in indigo B [1.241(3) Å]. In the molecular structure of **3**, the chiral C13­(R) and C14­(R) carbons have hydrogen, and the C13–C14 [1.546(3) Å] bond distance is significantly extended. The Boc and indolin-3-one groups are also folded over each other. The C–N bond length in the indolin-3-one is in the range of 1.412(3)–1.462(3) Å. As in example **2**, these distances are longer than in the indigo B molecule.^21^ Similarly, the carbonyl lengths [C12=O3 1.215(3) Å and O4=C15 1.206(3) Å] in **3** are shorter. All of these changes are attributed to the electron distribution and conformational effects induced by the Boc substitution Figure 5.

**Figure 5:**
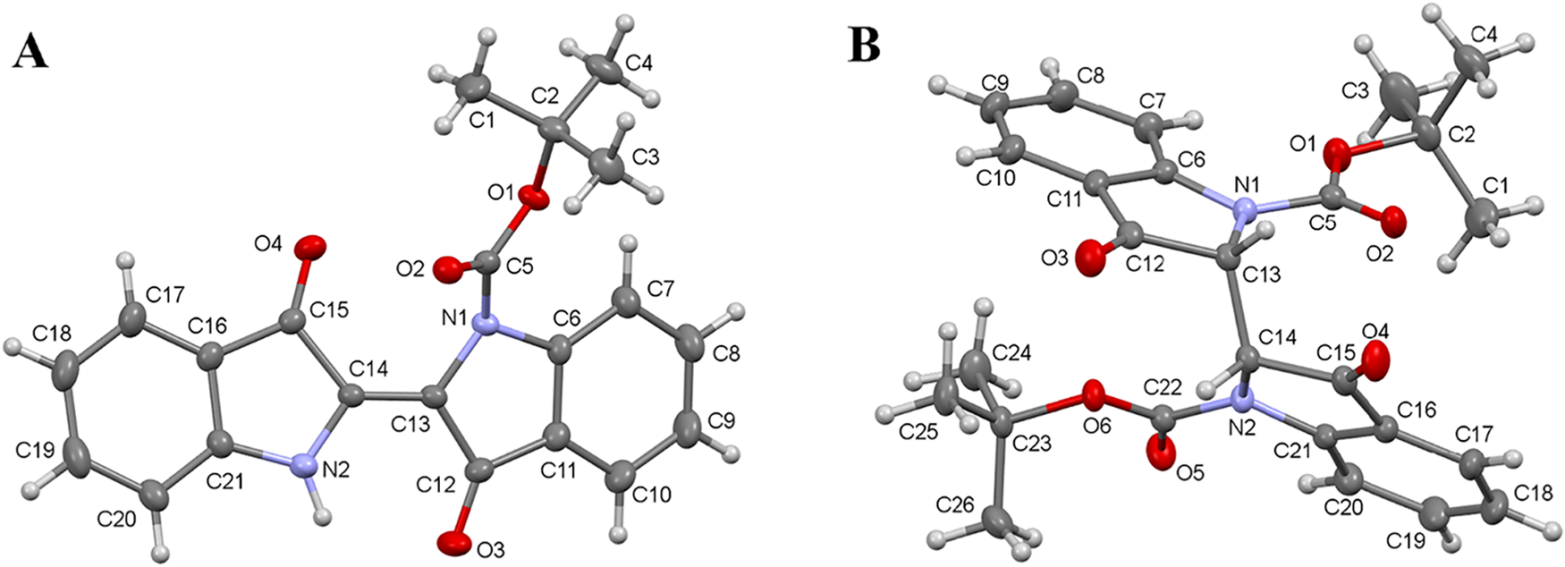
Molecular structure of **2** (A) and **3** (B). Thermal ellipsoids are given at the 40% probability level. The unit cell with the crystal lattice and X-ray data for each molecule is given in the Supporting Information section.

### Photophysical Properties

The absorption and emission properties of all compounds in various solvents were evaluated, and their molar absorption coefficients were determined (Table 1). Several studies in the literature have been reported on the photophysical properties of **1**, including absorption behavior and *E–Z* isomerization;^4b,5a,16a,22^ however, its solid-state fluorescence property is disclosed for the first time in this study (Figure 6). Dye **1** exhibited emission maxima at 650 nm, and the red crystals appeared bright yellow under UV light. This fluorescence property was also observed on both natural and synthetic textiles dyed with **1**, indicating that **1** can potentially be used as a tracer/taggant for natural and synthetic textile materials (Figure S11 in the Supporting Information). Furthermore, we observed that the fluorescence is quenched significantly in organic solvents and even on a silica-coated TLC plate. Moreover, absorption and emission spectra of **1** in different solvents were evaluated (Figure S4 in the Supporting Information).

**Figure 6:**
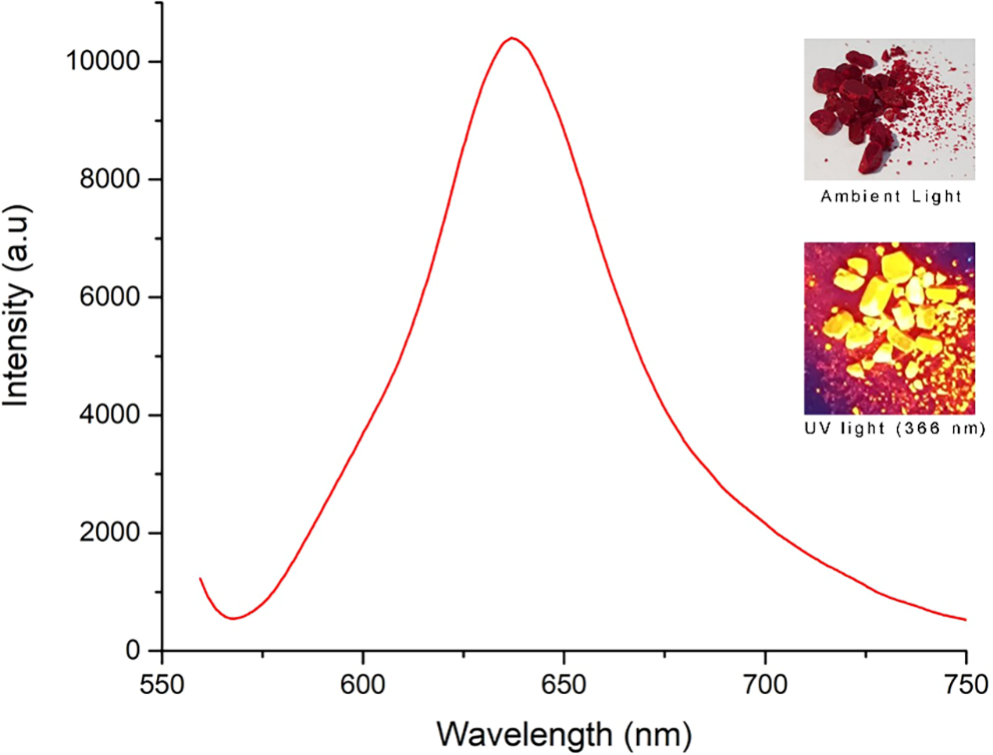
Solid-state fluorescence spectrum of **1** and its images under ambient and UV light (λ_ex_: 550 nm, λ_em_: 650 nm).

**Table 1:** Absorption and Emission Properties of **1**, **2**, and **3** in Different Solvents

	**1**		**2**			**3**	
Solvents	λ^a^_max. abs._ (nm)	ε (cm^–1^ M^–1^)	λ^a^_max. abs._ (nm)	λ^b^_max. em._ (nm)	ε (cm^–1^ M^–1^)	λ^a^_max. abs._ (nm)	λ^b^_max. em._ (nm)
Toluene	546	73203	556	371, 644	128490	340	386
THF	544	78209	558	370, 646	117497	340	392
CHCl_3_	546	90439	558	377, 656	163459	344	400
DCM	546	85131	556	374, 656	169990	342	397
Acetone	542	81371	554	407, 650	144864	340	394
DMSO	548	78162	572	374, 680	118754	342	398
MeOH	544	81928	562	389, 668	140376	342	422

a Long wavelength absorption maximum, in nm; *c* = 60 μM.

b Fluorescence maximum, in nm; *c* = 10 μM; λ_ex._= 330 nm for **2** and λ_ex._= 325 nm for **3**.

As previously reported by Melo et al.,^16c^ we similarly observed that **2** shows a slight solvatochromic behavior with increasing solvent polarity, accompanied by a relatively low fluorescence intensity in different solvents (Figure S9 in the Supporting Information). Distinct from previous studies on **2**, we further investigated its deprotonation behavior at different pH in DMSO–Britton–Robinson buffer mixture (9:1, v/v) and observed significant bathochromic shifts of 80 and 188 nm in the absorption spectrum from the maximum at 318 to 398 nm, and from the maximum at 572 to 760 nm, respectively (Figure 7). Three isosbestic points at 350 nm, 460 and 630 nm were also observed. In addition, because of the keto–enol tautomeric mixture of **2**, there are two possible alternative forms for the deprotonated structure of **2**. Density functional theory (DFT) calculations were performed to evaluate the expected charge increase on the O3 and N2 atoms (Figure 8) after deprotonation at the N2 position. The results show that the charge on O3 increases from −0.63534 to −0.68608 (an 8% increase), while the charge on N2 increases from −0.56327 to −0.62322 (an 11% increase).

**Figure 7:**
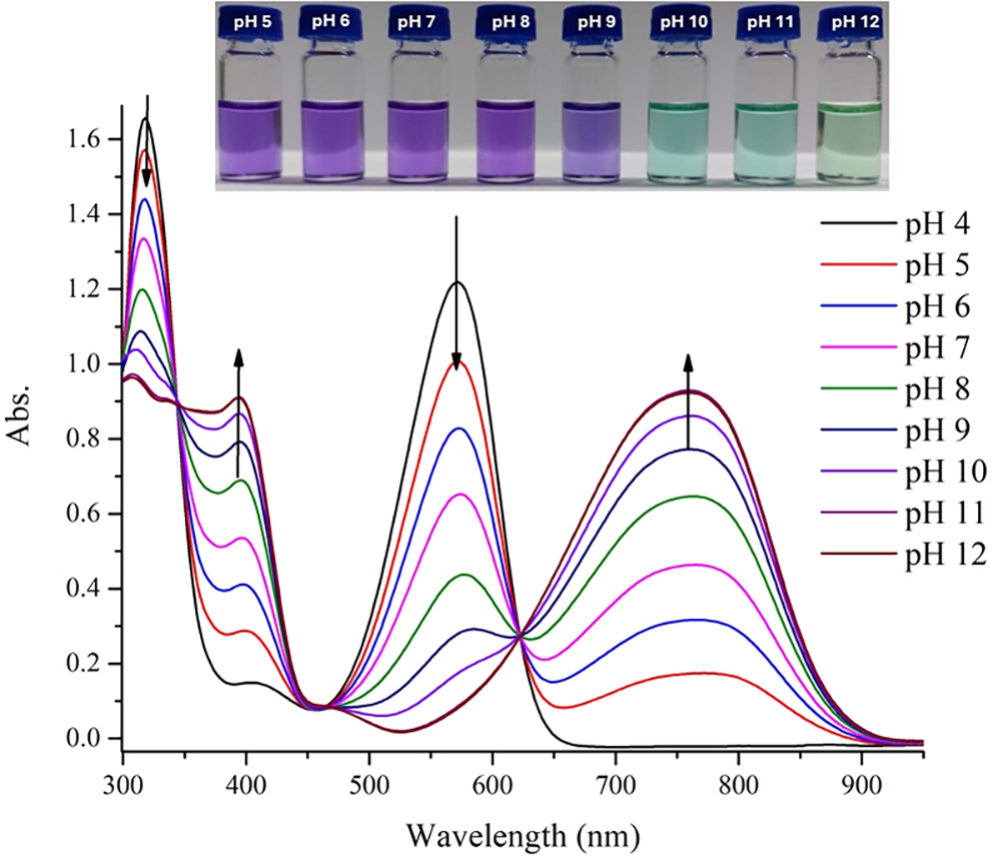
Absorption spectra of **2** (*c* = 100 μM) at different pH in DMSO–Britton–Robinson buffer mixture (9:1, v/v).

**Figure 8:**
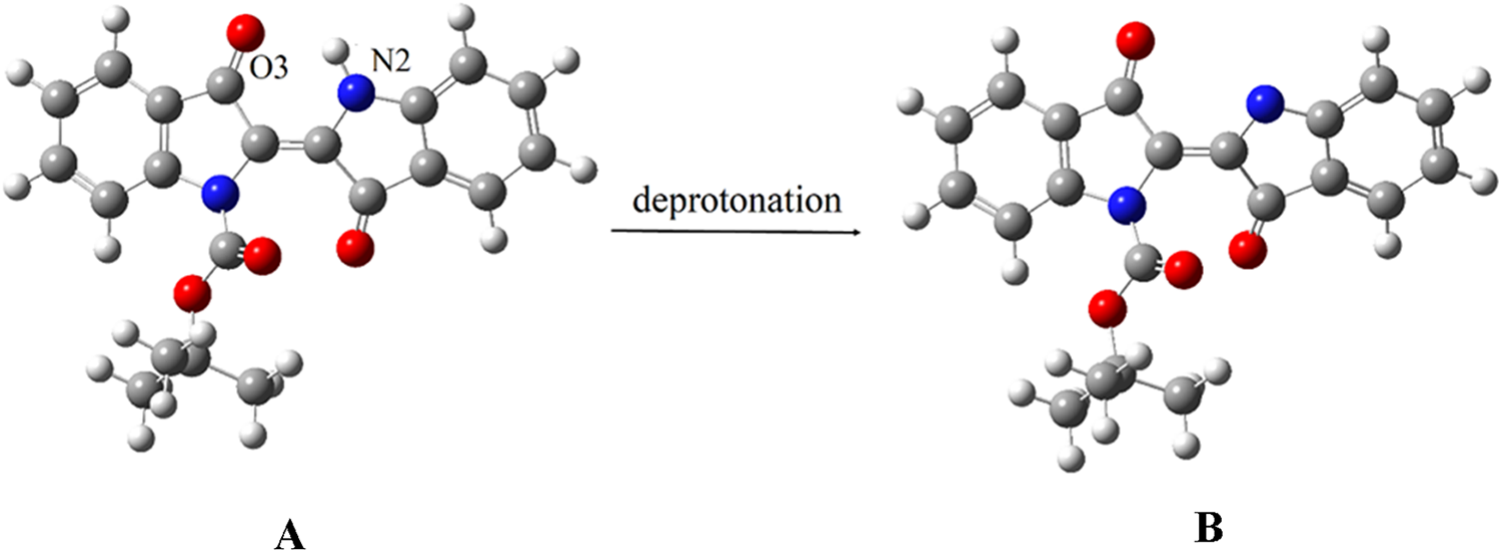
Optimized geometries of **2** (A) and its deprotonated forms (B) at the B3LYP/6–311+G­(d,p) level.

The photophysical behavior of **3** was investigated, and its absorption and emission wavelengths are shown in Table 1. It is colorless; however, fluorescence emission wavelengths were observed in the near-visible region, with a range of 386–400 nm, in all solvents (except methanol) used. And, it showed a significantly higher fluorescence intensity observed in the protic solvent methanol at 422 nm (Figure S10 in the Supporting Information).

### Theoretical and Experimental Studies on the Proposed Oxidation Mechanism of **3**

We observed that the conversion of **3** to **1** occurs via an oxidation reaction in the presence of NaOH under oxygenated conditions. To gain further insight into this conversion, the same reaction was carried out under an inert atmosphere. The solution was purged with nitrogen gas for two hours. After the addition of 0.1 equiv of NaOH during the purging process, the originally colorless solution turned pale yellow. This color remained unchanged until the system was exposed to the atmosphere. Notably, upon exposure to air, the color rapidly changed from yellow to magenta, indicating the progression of the oxidation reaction.

In the proposed reaction mechanism, the transformation is initiated when the molecule adopts a specific conformation, defined as rotational isomer A (Figure 9, **3a-TS1**), in which both nitrogen atoms are positioned on the same side of the molecular framework. This spatial arrangement enables the hydroxide ion (OH^–^) to abstract a proton from an sp^3^-hybridized carbon atom, leading to the formation of a carbanion intermediate (**3a**) via the transition state **3a**-**TS1**. Following this step, water is eliminated through an interaction with the carbanion intermediate (**3b**). The removal of water enables delocalization of the negative charge via resonance from the carbon atom to the oxygen atom, leading to the formation of a five-membered ring system, which is calculated as the transition state, via a hydrogen bond (**3c-TS2**). Subsequently, a structural rearrangement occurs, giving rise to a seven-membered ring system through a hydrogen-bonding interaction between the two oxygen atoms, stabilized by resonance. The total energy of the system decreases as the reaction proceeds through the transition states, eventually reaching −17.7 kcal/mol relative to the starting molecule under oxygen-free conditions (Figure 9A).

**Figure 9:**
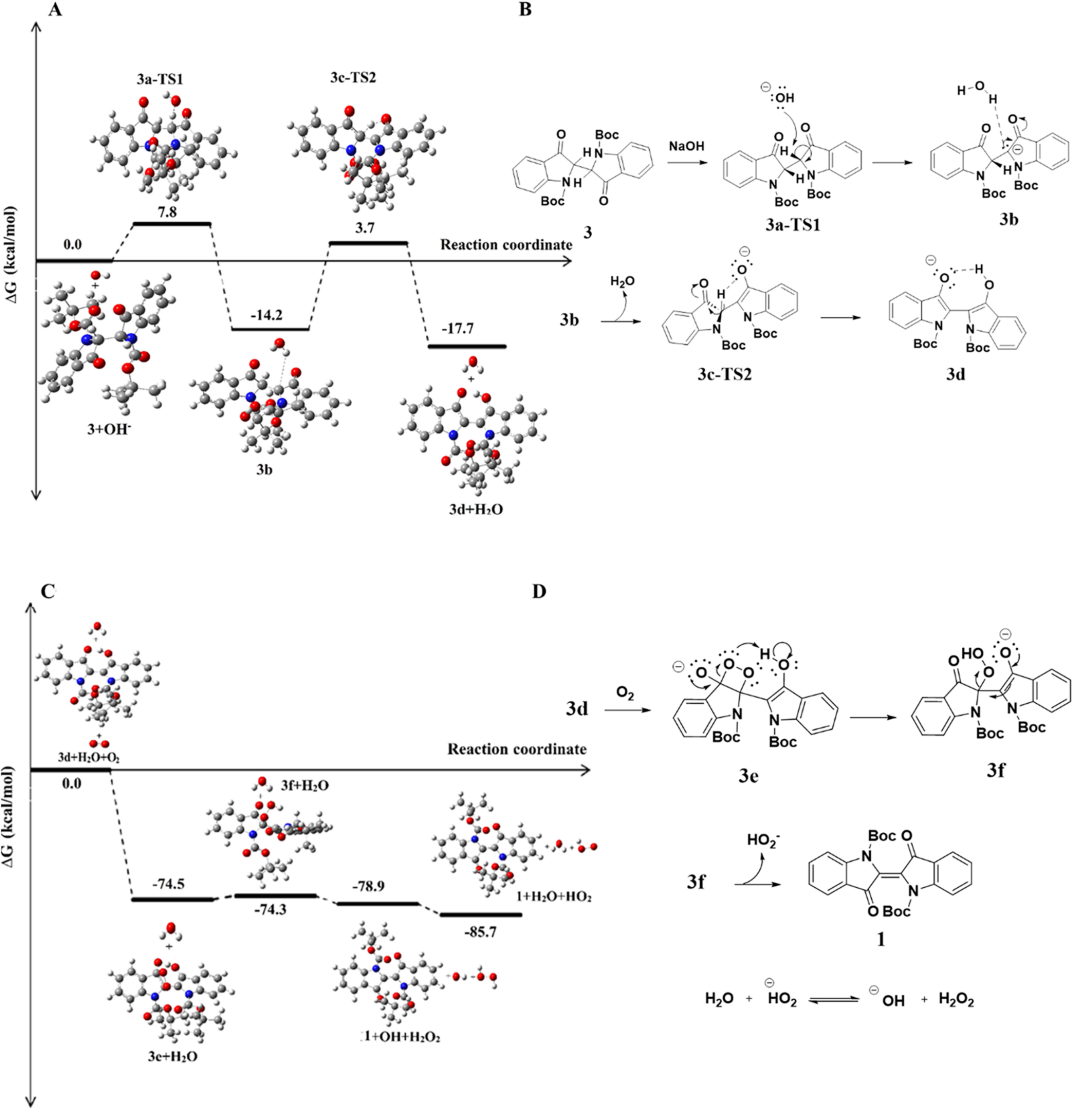
Reaction coordinate diagram in gas phase and DMSO at the B3LYP/6–311+G­(d,p) level, (A, B) for the deprotonation of **3** by OH^–^ to **3d** calculated, (C, D) Reaction coordinate diagram for the reaction of **3d** by O_2_ to **1** and elimination of a hydroperoxide anion.

When the system is exposed to air, singlet oxygen (^1^O_2_) rapidly undergoes a [2 + 2] cycloaddition reaction with the C=C double bond of the enolate, resulting in the formation of a dioxetane intermediate.^24^ In this structure, bond lengths between oxygens and carbon atoms were calculated to be 1.471 and 1.530 Å, respectively (Figure 10).

**Figure 10:**
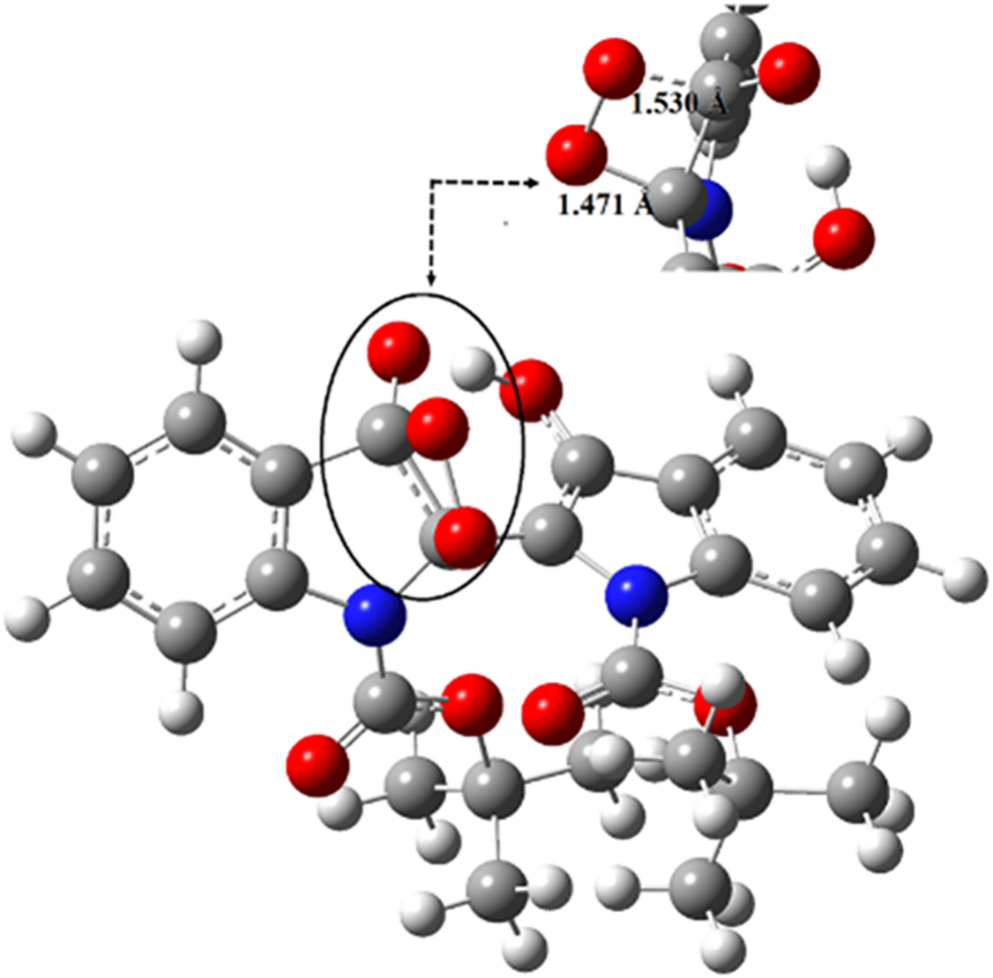
Molecular structure of dioxetane intermediate (**3e**) and bond lengths between oxygen and carbon atoms at the B3LYP/6–311+G­(d,p) level.

**3e** undergoes a rearrangement following the abstraction of a hydrogen atom from the other hydroxyl group of enol, resulting in the formation of the molecule **3f**. This transformation, reminiscent of the interaction of reduced flavin with molecular oxygen to form (hydro)­peroxoflavin intermediates, leads to the formation of an organic peroxide species.^24c^ The energy level of this oxygenated intermediate is significantly lower (−92.2 kcal/mol) than that of the initial **3**, indicating a thermodynamically favorable oxidation process. Finally, the reaction concludes with the elimination of a hydroperoxide anion (HO_2_^–^), which reacts with water to generate hydrogen peroxide (H_2_O_2_) and a hydroxide ion (OH^–^). The overall reaction is driven by a significant thermodynamic gradient, with a net energy change of −103.4 kcal/mol from **3** to the final product **1**, which strongly favors product formation (Figure 9C). In addition to the experimental and theoretical studies, ^1^H NMR titration experiments with tetrabutylammonium hydroxide (TBAOH) in DMSO-*d*_6_ were performed to further support our observations (Figure 11).

**Figure 11:**
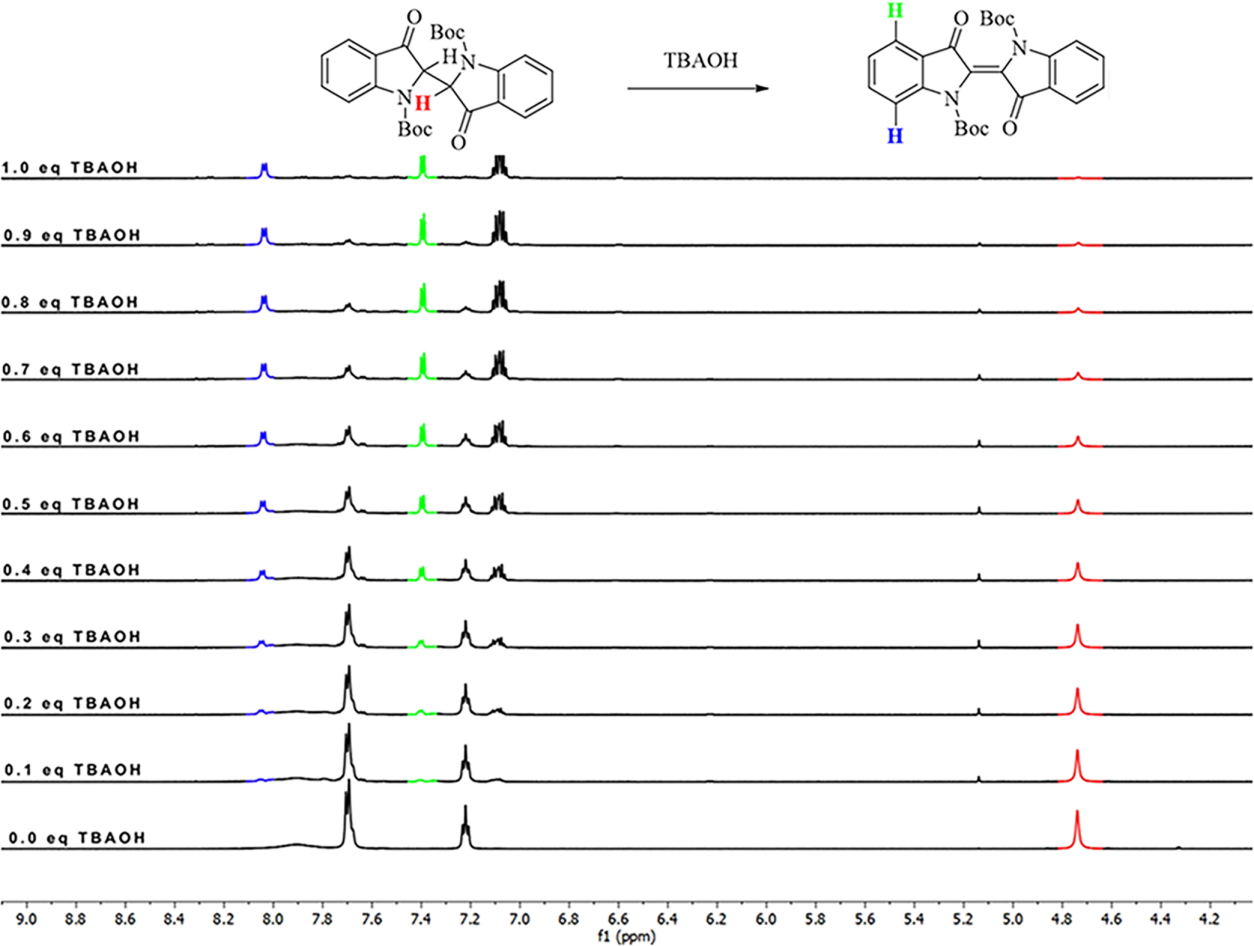
^1^H NMR (600 MHz) titration studies with TBAOH in DMSO-*d*_6_.

The disappearance of the proton signals corresponding to the acidic proton at approximately 4.7 ppm, along with the appearance of new signals at 8.1 and 7.4 ppm in the ^1^H NMR spectrum, confirmed the oxidation of **3** to **1** in the presence of hydroxide ions under atmospheric conditions. Furthermore, the ^1^H NMR titration results indicated that a stoichiometric amount of TBAOH is required for the completion of the oxidation reaction, as shown in the proposed mechanism.

For further confirmation of obtaining **3** from **1**, a UV/vis titration study was also performed. Compound **3** is colorless, and its absorption maxima in all solvents used were observed at the UV region with the range 340–342 nm (Table 1 and Figure S6 in the Supporting Information). Therefore, its spectrum in DMSO was not included in the visible spectrum of the titration. This study (Figure 12A) demonstrated that **3** can be converted back to **1** through an oxidation reaction involving the gradual addition of NaOH in DMSO at room temperature. Furthermore, **3** can also be converted to the deprotonated form of **2**, depending on the amount of NaOH under the same conditions. After the addition of HCl acid, the deprotonated form of **2** reverts to **2** (Figure 12B).

**Figure 12:**
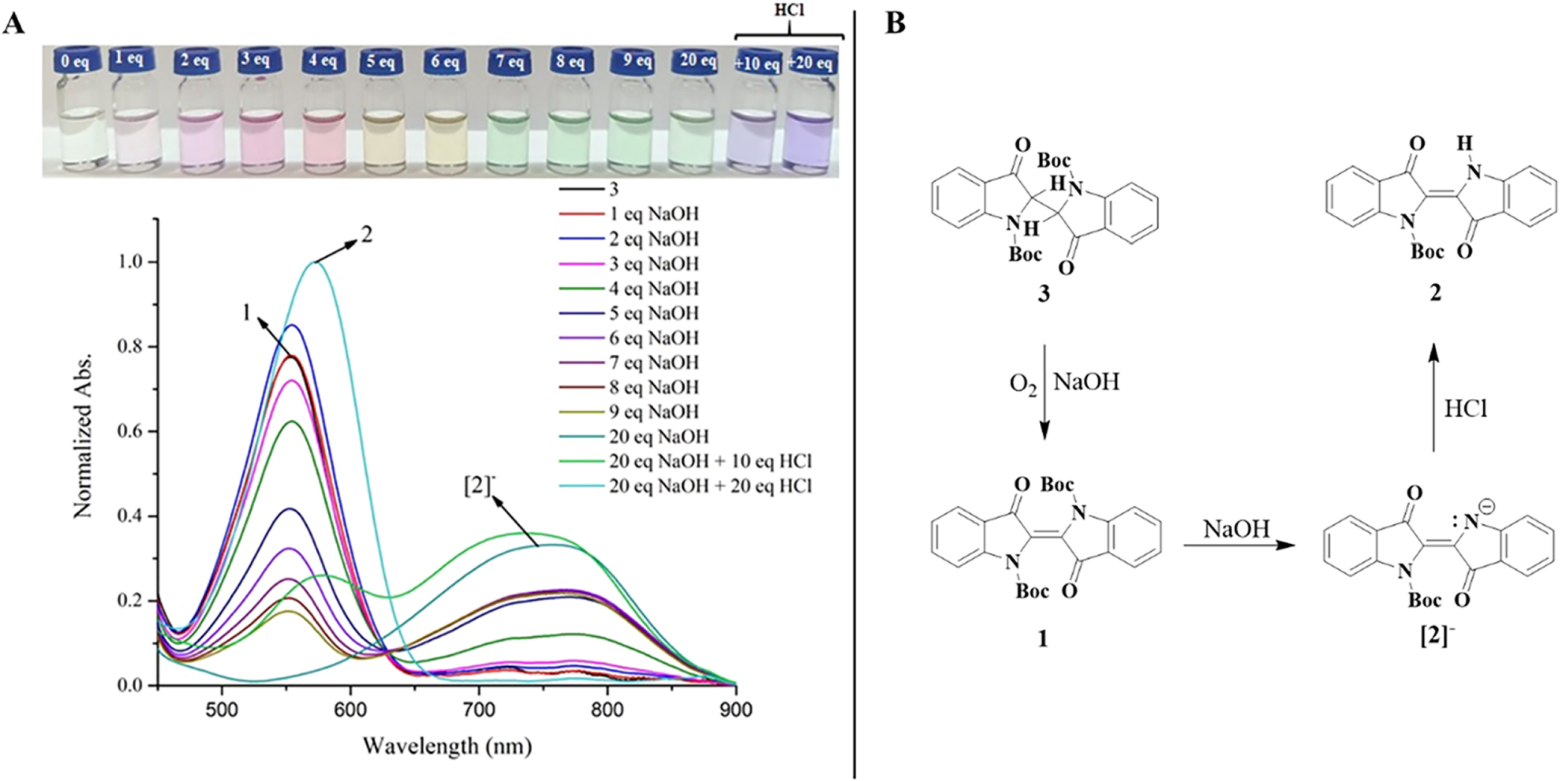
UV/vis titration studies (A) (*c* = 60 μM) in DMSO and conversion reaction pathway of **3** to **2** (B).

### Thermal Conversion

The Boc group is known to be a common thermal labeling group and is used in *N*-protection chemistry.^4a,16a,23^ We investigated the thermal properties of all synthesized Boc-substituted compounds by TGA and compared them with those of unsubstituted indigo. Our results demonstrated that **1**, **2**, and **3** undergo thermal degradation via unsubstituted indigo (Figure 13A). Additionally, **1**, **2**, and **3** are degraded on silica-coated plates at relatively high temperatures (200–450 °C) (Figure 13B). These findings indicate that the conversion of **3** to unsubstituted indigo occurs via oxidation and degradation/deprotection reactions, respectively. Since the oxidation reaction occurred so rapidly, the conversion of **3** to **2** was not visually detectable.

**Figure 13:**
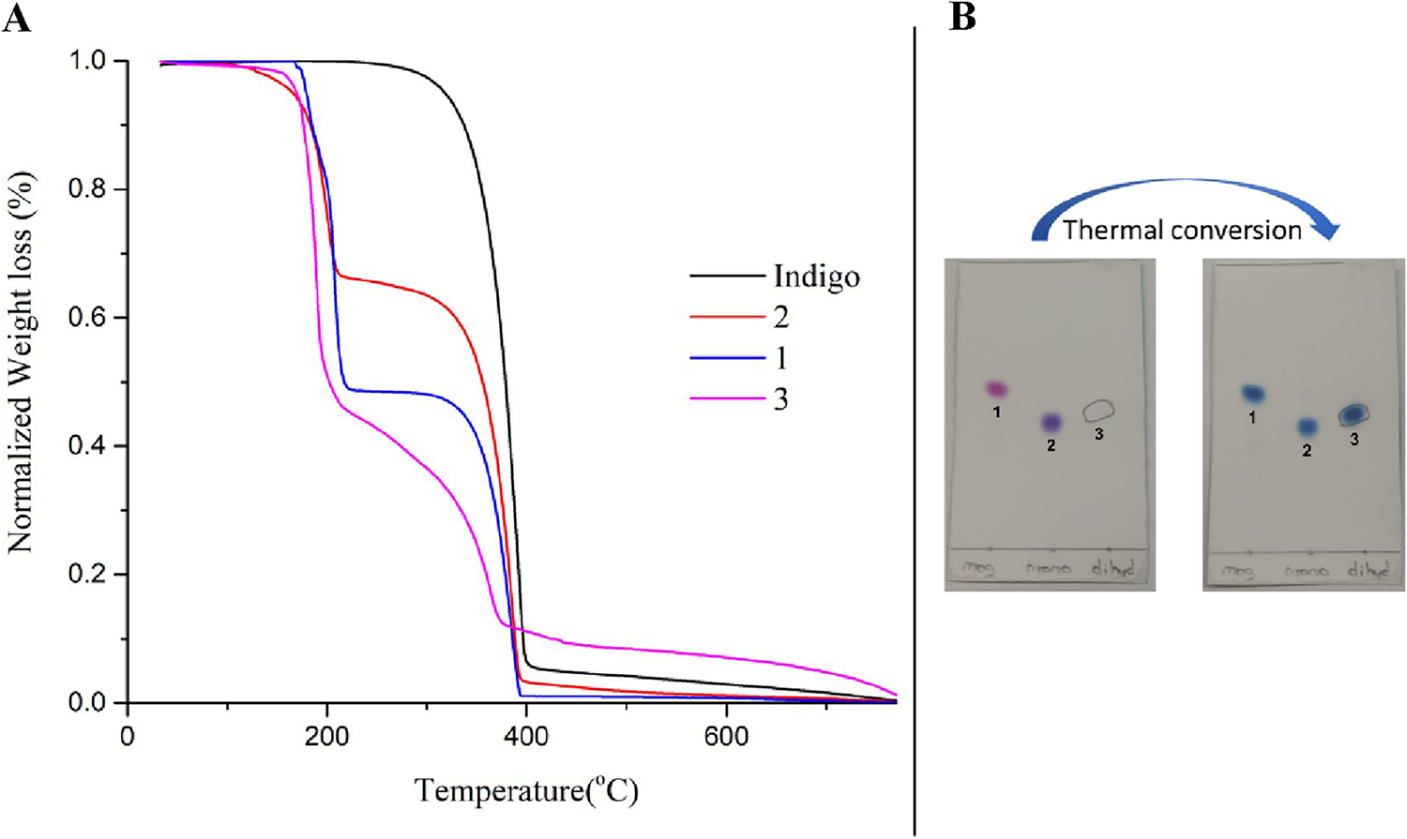
Thermal decomposition of indigo, **1**, **2**, and **3** (A: in inert condition (10 °C/min), B: on a TLC plate).

### Textile Dyeing and Thermal Treatment

The preliminary studies on the use of **1** as a dye for textiles in the conventional vat dyeing process were patented by our group.^6a^ In this study, we further explore its potential in textile dyeing processes. Since synthetic textiles cannot be dyed with indigo using conventional methods, specific approaches are required. These include the use of high temperatures and high pressure,^25^ special dye dispersion techniques,^26^ the use of additives such as acids and urea,^27^ appropriate reducing agents,^28^ controlled pH values,^29^ optimized dyeing and oxidation times,^30^ and pretreatment processes prior to dyeing.^31^ In contrast to the conventional indigo dyeing process, synthetic fibers are dyed at acidic pH due to the anionic repulsion between the polymer chains of the textiles and the reduced indigo dye molecules.^3b^ However, we demonstrated that synthetics can be dyed with indigo derivatives in the conventional process at high pH and room temperature (Figure S19 in the Supporting Information). The Boc groups enhance the affinity of the dye molecules for synthetic fibers, PET, PP, and PU (Figure 14).

**Figure 14:**
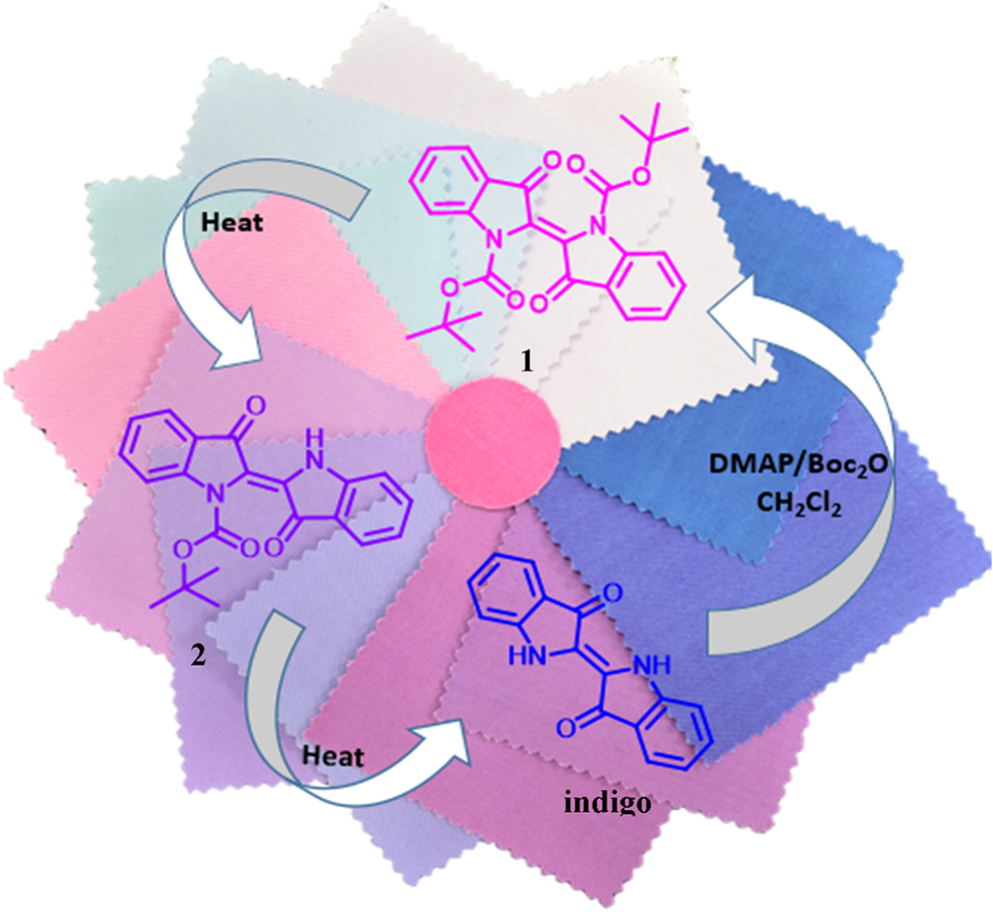
Dyed textiles (PET, PP, and PU) with **1** and their color spectra after thermal treatment.

After dyeing, the thermal labeling group is removed by heat treatment, yielding **2** and an unsubstituted indigo on the textiles, respectively. By using this approach, a range of colors can be obtained from magenta (**1**) to indigo blue. In addition, this method offers significant advantages, including reduced water and energy consumption, less labor, and faster access to a variety of color outcomes, an essential advantage for the dynamic demands of the fashion industry.

The standard color fastness tests were conducted in accordance with the relevant ISO methods, and the results were generally promising. As expected, the ratings for light fastness, dry cleaning, and oxidative bleaching were below grade 3 (Table S1 in the Supporting Information). Despite these limitations, the inherent characteristics of dye molecules offer considerable potential for fashion designers, particularly in the development of innovative and futuristic garment designs in denim applications.

## Conclusion

In conclusion, we have shown that the synthesis of **1** on a kilogram scale is possible without further purification and, at the same time, demonstrated the usability of textile waste dyed with indigo as a sustainable source of indigo. The ability to strip dyes from postindustrial textile waste and convert them into value-added chemical compounds holds significant potential not only for textile-to-textile recycling but also for broader chemical recycling strategies.

Furthermore, the selective synthesis of **2** from **3** at room temperature was achieved in a very short time via a single-step deprotection reaction. In view of the widespread use of indigo derivatives in various functional applications, the direct synthesis of novel indigo-based compounds continues to be of great importance. In this context, **2** serves as a versatile and valuable intermediate for further derivatization reactions and the development of functional materials.

This study also represents the first successful synthesis of **3**, which is a reduced form of **1** under neutral pH conditions, in contrast to previously known methods. Its molecular structure was fully elucidated by single-crystal X-ray diffraction, revealing a thermodynamic preference for the keto tautomer. To gain a deeper insight into its reactivity, the oxidation mechanism was investigated by combined experimental and theoretical approaches. These investigations revealed that the oxygen addition step takes place via [2 + 2] cycloaddition reaction and is followed to form **1**, which is very similar to the photo-oxidation of adamantylideneadamantane.^24b^

The application of **1** in the dyeing of synthetic fibers was successfully demonstrated and enabled the dyeing of PET, PP, and PU fabrics that cannot be dyed with indigo using conventional methods. Beyond its dyeing capability, the compound exhibits tunable thermal properties that result in a color palette ranging from magenta to indigo blue, increasing the functional versatility and aesthetic value. Due to these unique properties, the dye and the textiles treated with it hold great promise for various applications in the fashion and textile industries, offering innovative opportunities for both designers and end users. This multifunctionality is expected to attract considerable interest in the technical textiles market and in the high-end design sector.

## Experimental Section

### General Methods

Starting materials and reagents were commercially available and used as obtained from Sigma-Aldrich and Merck without further purification. Indigo used for rope dyeing was purchased from the Liyang company. Thin-layer chromatography (TLC) was used for monitoring the reactions by using precoated silica gel 60 F254 plates. NMR spectra were measured on a Bruker ASCEND 600 (^1^H: 600 MHz, ^13^C: 151 MHz) spectrometer at 25 °C. The ^1^H NMR and ^13^C­{^1^H}-NMR spectra were referenced to the residual proton signal of the solvent [DMSO-*d*_6_: δ­(^1^H) = 2.50 ppm, δ­(^13^C) = 39.52 ppm or CHCl_3_: δ­(^1^H) = 7.26 ppm, δ­(^13^C) = 77.16 ppm]. Chemical shifts (δ) are given in parts per million (ppm) using the residue solvent peaks as a reference relative to TMS. Coupling constants (*J*) are given in hertz (Hz). The splitting patterns are designated as follows: s (singlet); d (doublet); t (triplet); m (multiplet); dd (doublet of doublets); ddd (doublet of doublets of doublets); dt (doublet of triplets); td (triplet of doublets); and br (broad). High-resolution mass spectra (HRMS) were recorded using Agilent Technologies 6530 Accurate-Mass Q-TOF LC/MS. Absorption and emission spectra were recorded on a Horiba Duetta spectrophotometer at 25 °C. FTIR (ATR) spectra of the compounds were taken with a PerkinElmer 100 FTIR spectrometer in the scanning range of 400–4000 cm^–1^. Thermogravimetric analysis (TG/DTA) measurements of the compounds were made using the HITACHI STA 7300 TG/DTA thermal analyzer device under a nitrogen atmosphere of 30–800 °C (10 °C min^–1^).

All geometry optimizations without symmetry restrictions were performed using density functional theory (DFT)^32,33^ with the B3LYP^34–38^ hybrid functional with the 6–311+G­(d,p) basis set. Frequency calculations at the same level were performed to ensure the local minima or the transition states and to derive the thermochemical corrections for the enthalpies and free energies. The solvent effect was considered using the integral equation formalism of the Polarizable Continuum Model (PCM)^39,40^ in DMSO. The transition states were estimated using the STQN method and verified by performing frequency calculations (only one imaginary frequency). In all energy diagram figures, calculated relative Gibbs free energies (kcal/mol) are presented. All of these calculations were carried out using the Gaussian16 program.^41^

The X-ray analyses of compounds were done on a Rigaku R-AXIS RAPID-S diffractometer (equipped with a two-dimensional area IP detector). Graphite-monochromated Mo–Kα radiation (λ = 0.71073 Å) and oscillation scan techniques with Δw = 5° for one image were used for data collection. Detailed information on methods can be found in the Supporting Information.

### Synthesis

#### Synthesis of *N*,*N*′-Di­(t-butoxycarbonyl)­indigo **(1)** on kg Scale

The indigo used in the synthesis was sourced from China (the name of the supplier is not disclosed due to commercial confidentiality). A total of 2.62 kg (10 mol) of indigo was ground into a fine powder and used in this form. The powdered indigo was placed in a 500 L reactor, followed by the addition of 400 L of dichloromethane (DCM) via an explosion-proof pump. Subsequently, 8.25 kg (37.8 mol) of di-*tert*-butyl dicarbonate (Boc_2_O) and 1.89 kg (15.5 mol) of 4-dimethylaminopyridine (DMAP) were added to the reactor under continuous stirring with an impeller at 75–100 rpm and room temperature. After 24 h, the reaction was stopped, and the solvent was removed using a pilot-scale Buchi evaporator (brand and model to be specified). The evaporator was connected directly to the reactor via a suitable pipe system so that the reaction mixture could be transferred to the evaporation flask (Figure S1 in the Supporting Information). After removal of the solvent, the solid product was collected and successively washed with water, 1 M NaOH, 1 M HCl, and again with water. These washing steps were used as a purification method instead of column chromatography. The final product was then dried in an oven at 70–80 °C for 8 h, yielding 4.15 kg (8.98 mol) (90%) of the red-pink powder (Figure S2 in the Supporting Information).

^1^H NMR (600 MHz, CDCl_3_): δ 8.02 (d, *J* = 8.3 Hz, 1H), 7.76 (ddd, *J* = 7.6, 1.5, 0.6 Hz, 1H), 7.60 (td, *J* = 7.8, 1.4 Hz, 1H), 7.20 (td, *J* = 7.5, 0.9 Hz, 1H), 1.62 (s, 9H). ^13^C­{^1^H} NMR (151 MHz, CDCl_3_): δ 183.6, 150.0, 149.0, 135.8, 125.0, 124.1, 124.0, 122.9, 116.6, 84.5, 28.1.

The dichloromethane used in the synthesis was recovered and reused for up to four consecutive reaction cycles. However, after the fourth cycle, the accumulation of *tert*-butyl alcohol, formed as a byproduct during the reaction, interfered with the deprotonation step of indigo, resulting in a significant decrease in product yield (Figure S3 in the Supporting Information).

#### Synthesis of *N*,*N*′-Di­(t-butoxycarbonyl)­indigo **(1)** from Waste Textiles

An excess of (Boc)_2_O (2.62 g, 12 mmol) and DMAP (1.1 g, 9 mmol) were added to a reaction flask containing 150 mL of DCM. 1.57 g of postindustrial textile waste dyed with indigo (3% w/w indigo), the amount of indigo present on the waste textiles was determined by UV/vis spectroscopy using the following procedure: 1 g of waste textiles was placed into a 100 mL volumetric flask, and 100 mL of reducing solution was added. The mixture was kept until the reduction was complete, as indicated by the color change. A sample was then taken from the solution and analyzed by UV/vis spectroscopy using a calibration curve, which had already been prepared, and had previously been cut into small pieces (5 cm), and was added to the reaction mixture. The reaction was stirred for 24 h with a magnetic stirrer at room temperature. After completion of the reaction, all of the extractable indigo was successfully converted to **1**, with an isolated yield of about 50–55% (45 mg). The yield was determined using an in-house method in which the indigo content of the dyed ropes was quantified by reduction in the presence of a reducing agent and NaOH, followed by UV/vis spectrophotometric analysis.

#### Synthesis of *N*-Mono­(t-butoxycarbonyl)­indigo **(2)**

1 mmol (462 mg) of di-Boc indigo was dissolved in 35 mL of DMSO, and 3.5 mL of 1 M NaOH solution was added dropwise. After the addition of NaOH, the reaction mixture successively changed from pink to purple and then to green. After stirring for 20–25 min at room temperature, the reaction mixture was slowly added dropwise into 400 mL of 1 M HCl. After addition, a purple solid **2** began to precipitate. The precipitate was collected by vacuum filtration using a Büchner funnel and dried at 70 °C after washing with distilled water. The isolated product was obtained in a 76% yield (275 mg, 0.76 mmol). The product was crystallized in an ethyl acetate:hexane mixture (1:6, v/v) at 4 °C.

In the second approach employed for the synthesis of **2**, 10 mmol (4.62 g) of compound **1** was dissolved in 100 mL of DMF, and 30 mmol (1.17 g) of NaNH_2_ was added gradually under stirring. The reaction mixture was stirred for 10 min at room temperature. Within a few minutes, the color of the mixture changed from magenta to deep blue, accompanied by a noticeable increase in viscosity. After completion of the reaction, the mixture was carefully added dropwise into a 1 M HCl solution, resulting in the immediate precipitation of a purple solid. The solid was collected by filtration and then dried at 70 °C after washing with distilled water. The isolated product was obtained in 25% yield (0.9 g, 2.5 mol). ^1^H NMR (600 MHz, CDCl_3_): δ 8.00 (d, *J* = 8.4 Hz, 1H), 7.78 (d, *J* = 7.6 Hz, 1H), 7.69 (d, *J* = 7.5 Hz, 1H), 7.63–7.55 (m, 1H), 7.51–7.43 (m, 2H), 7.22 (t, *J* = 7.4 Hz, 1H), 7.07–6.96 (m, 2H), 1.61 (s, 9H). ^13^C­{^1^H} NMR (151 MHz, CDCl_3_): δ 188.0, 185.9, 151.4, 149.9, 149.6, 136.4, 135.4, 130.1, 125.1, 123.8, 123.7, 123.3, 121.8, 120.0, 116.8, 116.2, 112.1, 84.3, 28.1. HRMS (ESI) *m*/*z* calculated for C_21_H_19_N_2_O_4_ ([M + H]^+^): 363.1345, found: 363.1352.

#### Synthesis of *N*,*N*′-Di­(t-butoxycarbonyl)-dihydroindigo **(3)**

10 mmol (4.62 g) of **1** was added to 250 mL of water, followed by the addition of 60 mmol (1.23 g) of Na_2_S_2_O_4_. The pH of the reaction mixture was then adjusted to 7 by using 1 M NaOH. The mixture was stirred for 7–8 days at room temperature. The precipitated product (a white to off-white solid) was collected by filtration. During the filtration, the solid was first washed with a fresh reducing solution (containing the same amount of Na_2_S_2_O_4_), then washed with distilled water, and subsequently dried at 60 °C. The product was obtained in 54% yield (2.54 g, 5.47 mmol), and it was crystallized in isopropyl alcohol. ^1^H NMR (600 MHz, CDCl_3_): δ 7.97 (br, 1H), 7.74 (d, *J* = 7.6 Hz, 1H), 7.57 (t, *J* = 7.0 Hz, 2H), 7.14 (t, *J* = 7.4 Hz, 1H), 4.77 (s, 1H), 1.23 (s, 9H). ^13^C­{^1^H} NMR (151 MHz, CDCl_3_): δ 195.7, 153.0, 151.1, 136.6, 124.3, 124.1, 123.1, 116.5, 83.8, 64.7, 27.5. HRMS (ESI) *m*/*z* calculated for C_26_H_29_N_2_O_6_ ([M + H]^+^): 465.2026, found: 465.2043.

## Supplementary Material









## Data Availability

The data underlying this study are available in the published article and its Supporting Information. The videos about the following syntheses and applications can be found in the links below and the Supporting Information section; synthesis of *N*,*N*′-di­(boc)­indigo, **1** from powder (mp4), 10.5281/zenodo.17381846; synthesis of *N*,*N*′-di­(boc)­indigo, **1** from waste textiles (mp4), 10.5281/zenodo.17381700.

## Notes

Dyeing of Textiles (100% PP, 100% cotton, 86% PET, and 14% Elastane) (MP4)
Color change of synthesized compounds on TLC sheet by thermal treatment (MP4)
Experimental and application procedures, absorption and emission spectra, computational and modeling results, application results, single-crystal X-ray structure analysis, NMR and mass spectra (PDF)
Proposed oxidation mechanism of **3** (MP4)
