# Supplementary material for: Indigo Beyond Tradition: Scalable Derivatization, Extraction from Waste Denim Textiles, and Boc-Protected Intermediates
Source: J Org Chem. 2025 Dec 31;91(2):1017–28. doi: 10.1021/acs.joc.5c02380 (PMC12814535; doi:10.1021/acs.joc.5c02380)
Supplement: Supplementary file 3 [file jo5c02380_si_003.pdf]

Supporting Information

Indigo Beyond Tradition: Scalable Derivatization, Extraction from Waste Denim Textiles, and Boc-Protected Intermediates

Gökhan Kaplan[a,b], Nurgül Seferoğlu[c], Ertan Şahin[d], Zeynel Seferoğlu*[e]

[a]Department of Chemistry, Graduate School of Natural and Applied Sciences, Gazi University, Yenimahalle, 06560 Ankara, Türkiye

[b]Sanko Tekstil İşletmeleri Sanayi ve Ticaret A.S. İsko Sb, Organize Sanayi Bölgesi 3. Cadde, 16400 İnegöl, Bursa, Türkiye

[c]Department of Advanced Technology, Graduate School of Natural and Applied Sciences, Gazi University, Yenimahalle, 06560 Ankara, Türkiye

[d]Department of Chemistry, Faculty of Science, Atatürk University, 25240 Erzurum, Türkiye

[e]Department of Chemistry, Faculty of Science, Gazi University, Yenimahalle, 06560 Ankara, Türkiye

***Corresponding Author,** Zeynel Seferoğlu; **Email:** znseferoglu@gazi.edu.tr

Keywords: Indigo, *N*,*N*'-mono/di(Boc)indigo derivatives, dihydro-di(Boc)indigo, oxidation, synthetics, dyeing, textile pollution.

Table of Contents

The set up for kg scale synthesis and image of synthesized product S3

Proposed reaction mechanism of synthesis of **1**....S4

UV/vis absorption spectra of **1**, **2** and **3** in different solvents....S5

Molar absorption coefficient measurement of **1** and **2**....S7

Fluorescence studies of **2** and **3** in different solvents....S9

Solid state fluorescence studies of dyed fabrics with **1**....S10

Single crystal X-ray structure analysis of **2** and **3**....S11

Mass spectra S16

IR spectra....S17

NMR spectra....S18

Dyeing studies of textiles S21

Colors of dyed textiles and color alterations after heat treatment S22

Color coordinates of dyed textiles and after thermal treatment S23

Color fastness of dyed fabrics S25

Cartesian coordinates of computed compounds in **Figures 4** and **11**....S27

Reference....S37

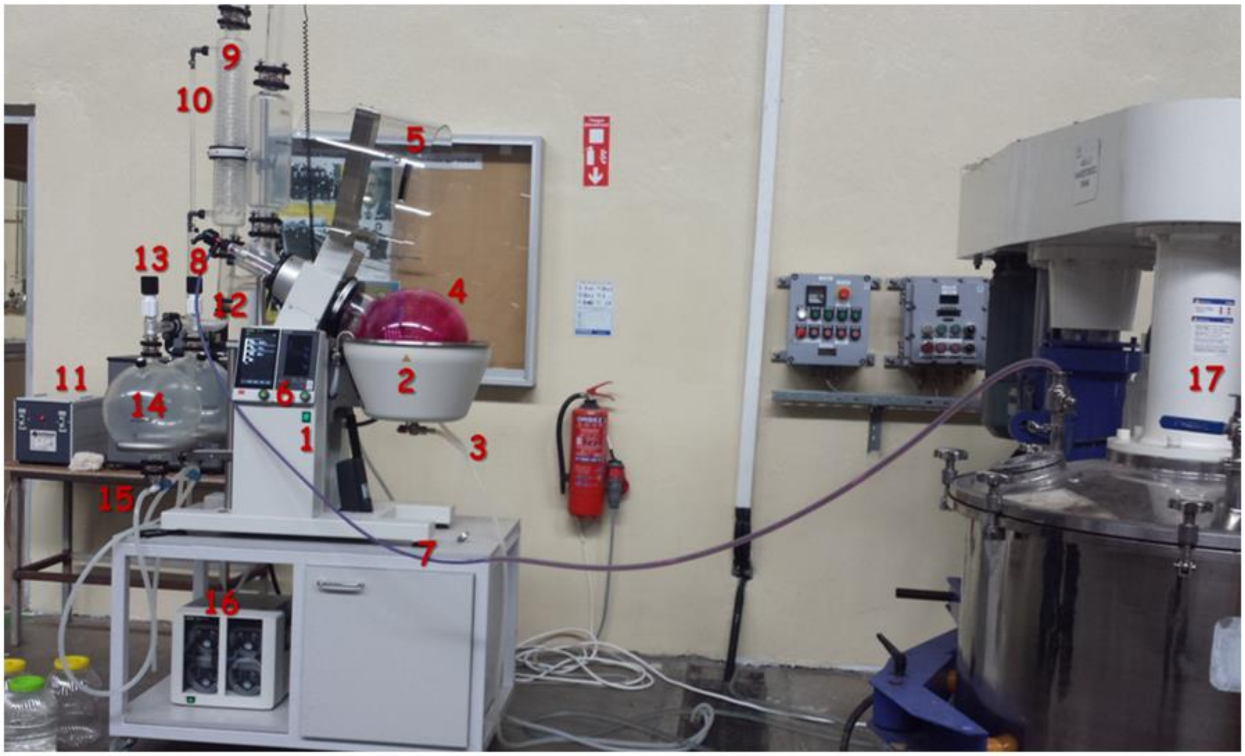

1 : Turn on/off button
2: Heating bath
3: Water inlet/outlet
4: Evaporating flask
5: Bath shield
6: Control panel / User interface
7: Pipe for the transfer from stirrer
8: Manual inlet valve
9: Condenser
10: Pipes for water circulation
11: Cooling circulation bath
12: Connectors
13: Shut-off tap
14: Receiving flask
15: Draining valve
16: Vacuum pump
17: 500 L industrial stirrer

Figure S1. The set up for kg scale synthesis.

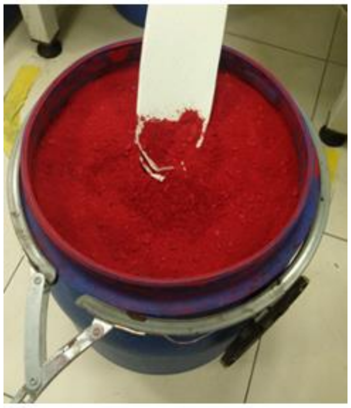

Figure S2. 25 kg of synthesized product.

Proposed reaction mechanism of synthesis of 1 and theoretical calculations

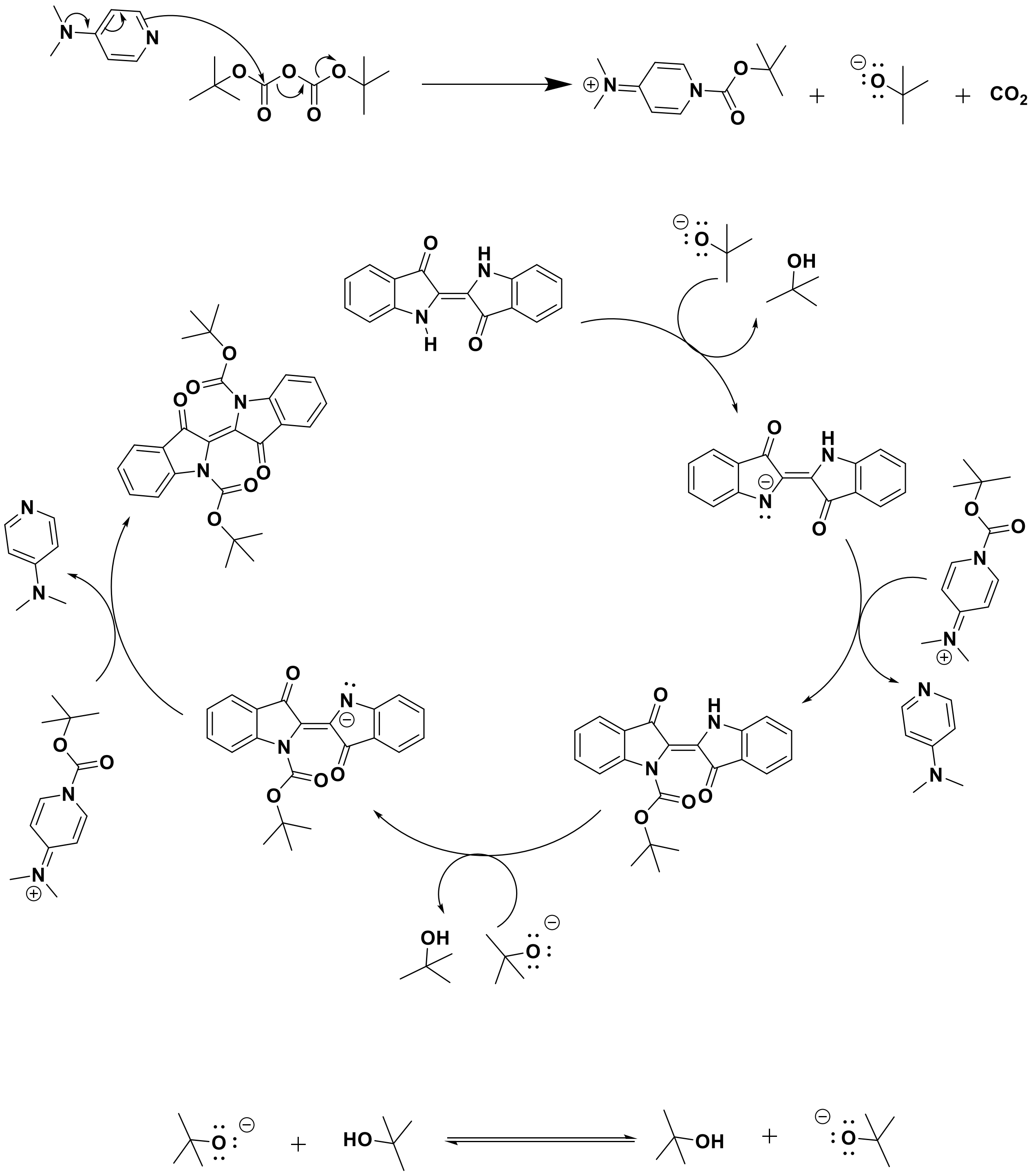

Figure S3. The proposed reaction mechanism of synthesis of **1**.

UV/vis absorption spectra of 1, 2 and 3 in different solvents

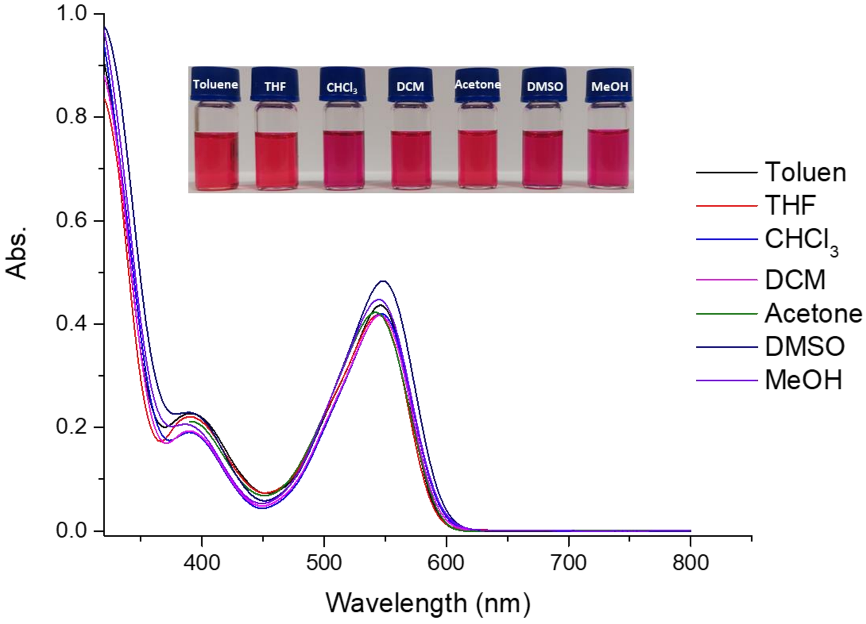

Figure S4. UV/vis absorption spectra of **1** in different solvents (*c*=60 µM).

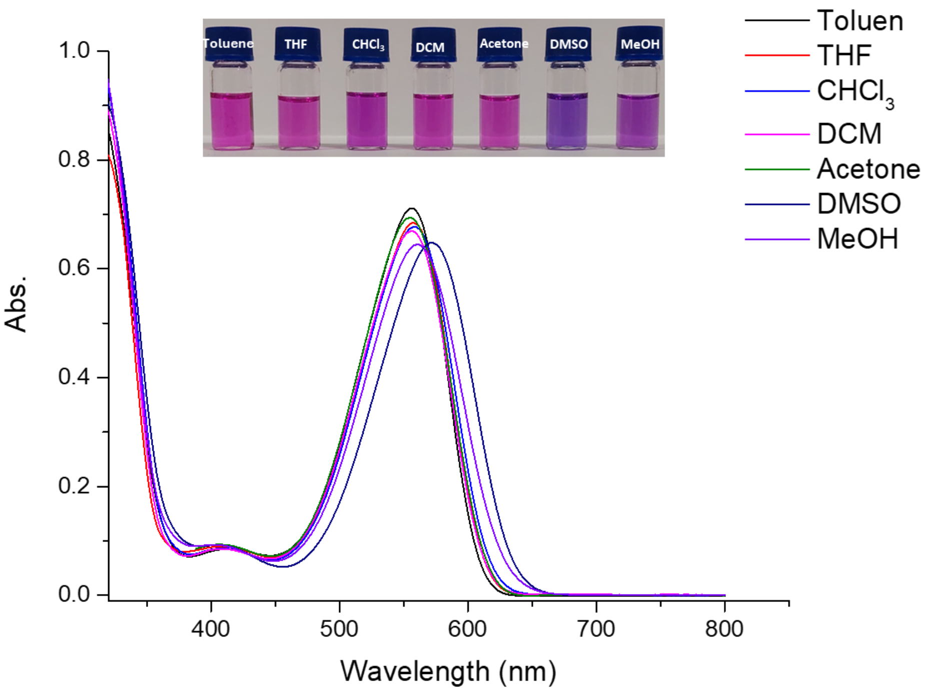

Figure S5. UV/vis absorption spectra of **2** in different solvents (*c*=60 µM).

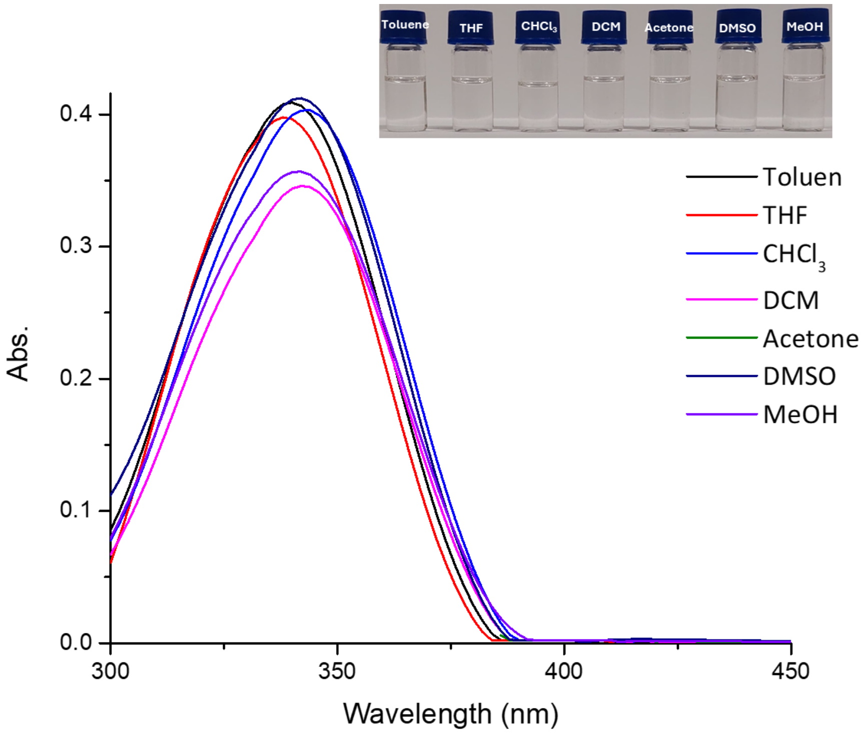

Figure S6. UV/vis absorption spectra of **3** in different solvents (*c*=60 µM).

Molar absorption coefficient measurement of 1 and 2

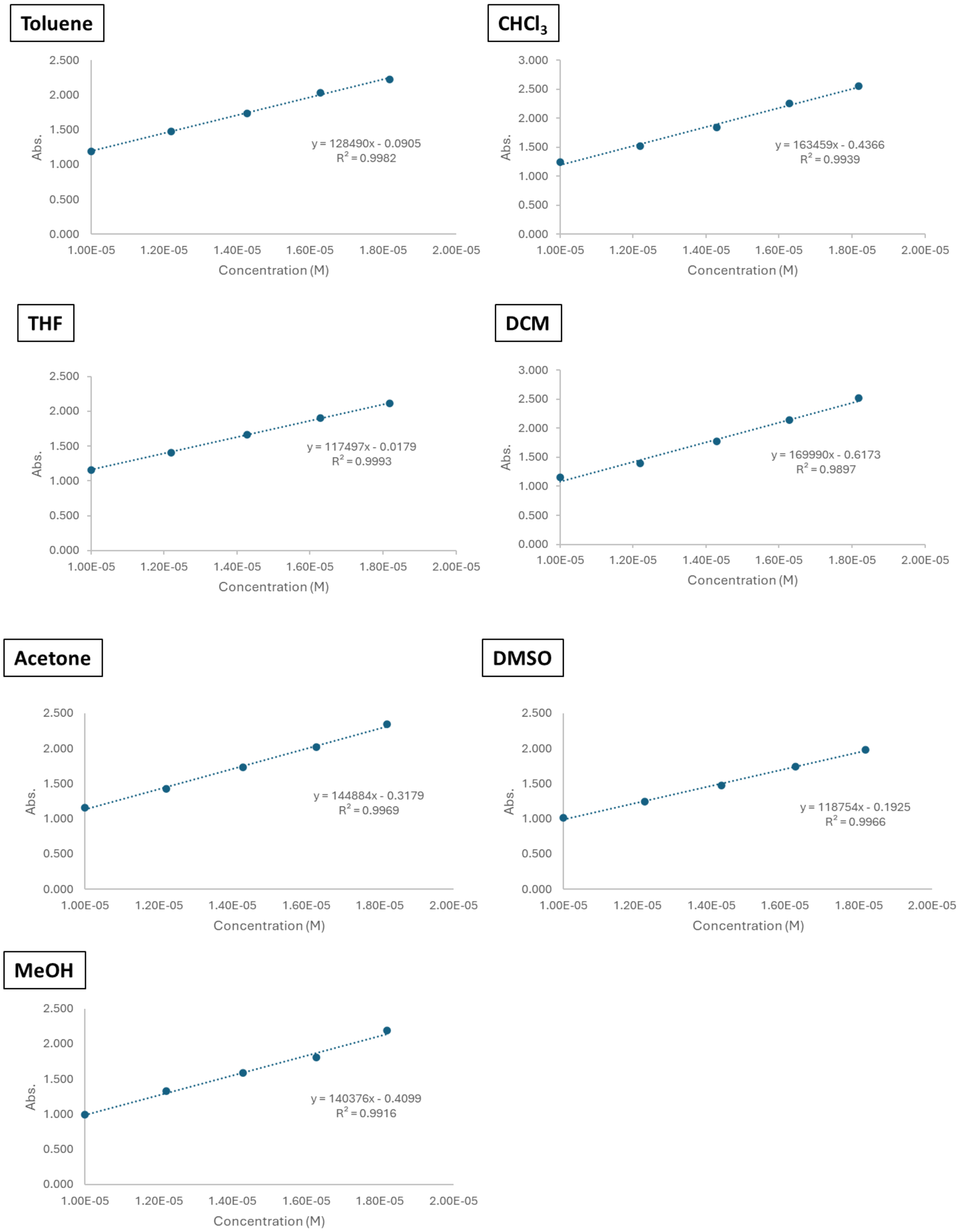

Figure S7. Molar absorption coefficient measurement of **1**.

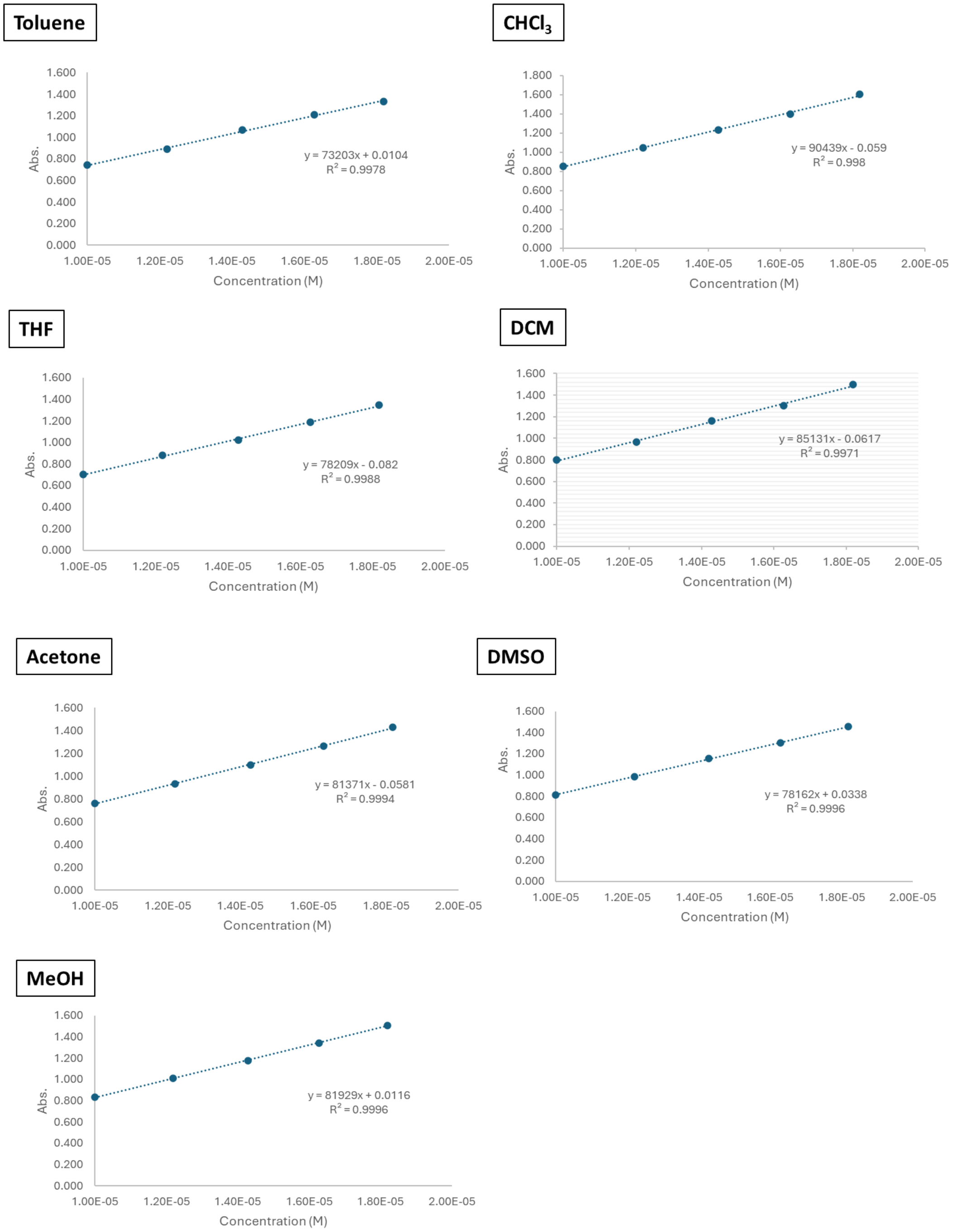

Figure S8. Molar absorption coefficient measurement of **2**.

Fluorescence studies of 2 and 3 in different solvents

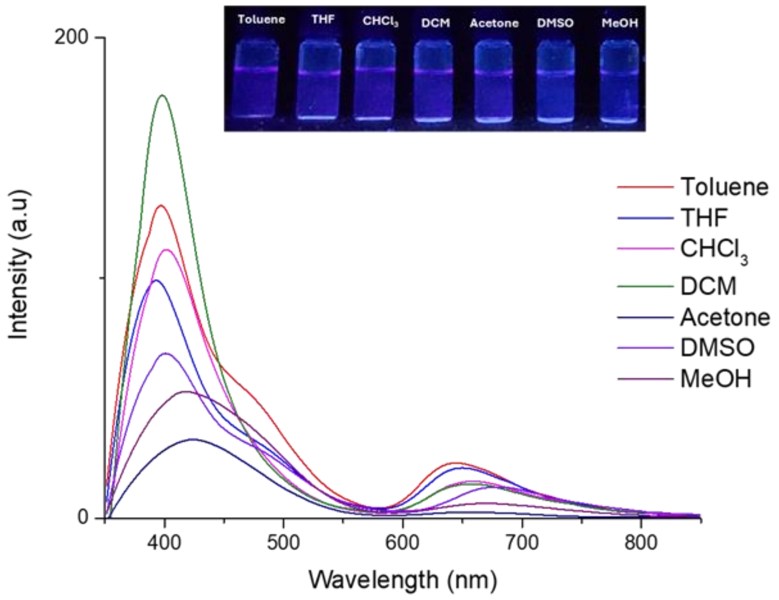

Figure S9. Fluorescence studies of **2** in different solvents (*c*= 10 µM, $\lambda_{ex.}$: 330 nm).

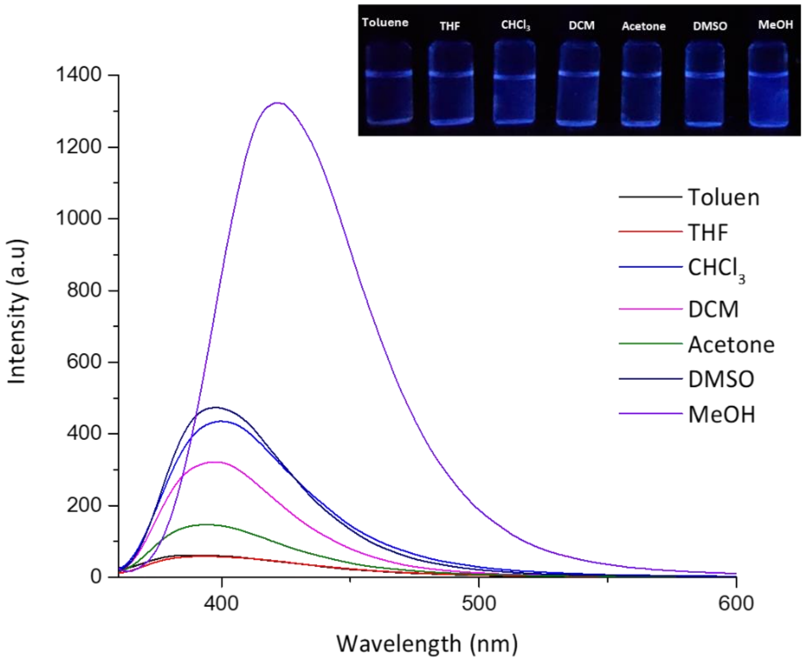

Figure S10. Fluorescence studies of **3** in different solvents (*c*= 10 µM, $\lambda_{ex.}$: 325 nm).

Solid state fluorescence studies of dyed fabrics with 1

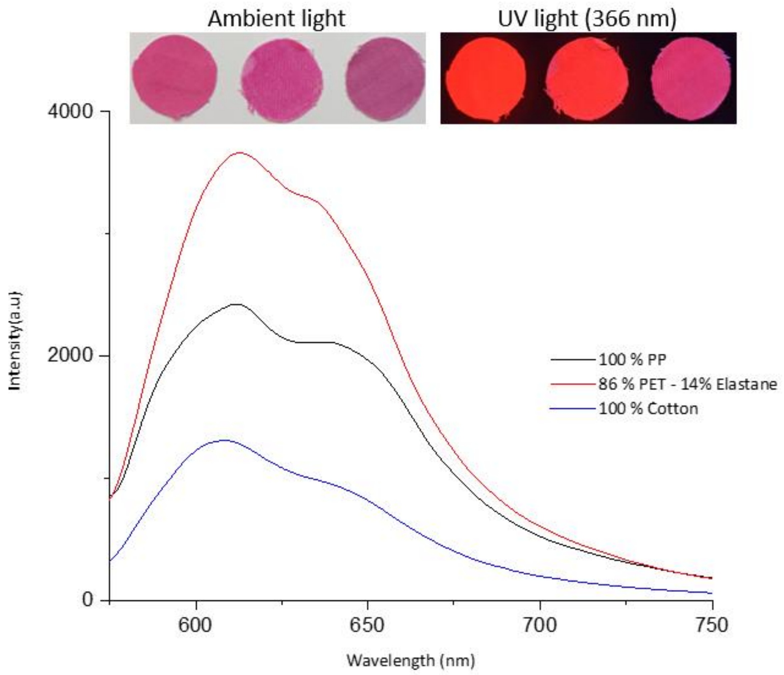

Figure S11. Solid state fluorescence studies of dyed fabrics with **1** ($\lambda_{ex.}$: 550 nm, $\lambda_{abs..}$: 610 nm) (fabric compositions from left to right 86 % PET-14 % Elastane, 100 % PP and 100 % Cotton).

Single crystal X-ray structure analysis of 2 and 3

The single crystals of **2** and **3** were grown from Ethyl acetate/Hexane and isopropyl alcohol solutions respectively at 4 °C. For the structure determination, single-crystals of t-butyl 3-oxo-2-(3-oxo-1,3-dihydro-2*H*-indol-2-ylidene)-2,3-dihydro-1*H*-indole-1-carboxylate (**2**) and di-t-butyl 3,3'-dioxo-2,2',3,3'-tetrahydro-1*H*,1'*H*-[2,2'-biindole]-1,1'-dicarboxylate (**3**) were used for data collection on a four-circle Rigaku R-AXIS RAPID-S diffractometer (equipped with a two-dimensional area IP detector). Graphite-monochromated Mo-Kα radiation (λ = 0.71073 Å) and oscillation scans technique with Δw = 5º for one image were used for data collection. The lattice parameters were determined by the least-squares methods on the basis of all reflections with F2>2σ(F2). Integration of the intensities, correction for Lorentz and polarization effects and cell refinement was performed using CrystalClear (Rigaku/MSC Inc., 2005) software. The structures were solved by direct methods using SHELXS-2013,[1] which allowed location of most of the heaviest atoms, with the remaining non-hydrogen atoms being located from different Fourier maps calculated from successive full-matrix least squares refinement cycles on F2 using SHELXL-2013.[1] All non-hydrogen atoms were refined using anisotropic displacement parameters. The hydrogen atoms were assigned with common isotropic displacement factors and included in the final refinement by using geometrical restrains. The final difference Fourier maps showed no peaks of chemical significance.

Crystal data for **2**: $C_{21}H_{18}N_2O_4$, crystal system, space group: orthorhombic, Pbca; (no:61); unit cell dimensions: a =11.7882(3), b = 8.2093(2), c = 37.1415(8) Å, α= 90, β= 90, γ = 90º; volume; 3594.3 (2) Å3, Z=8; calculated density: 1.339 g/cm^3; absorption coefficient: 0.094 mm^{-1}; F(000): 1520; θ-range for data collection 2.0-27.5º; refinement method: full matrix least-square on F2; data/parameters: 2540/248; goodness-of-fit on F2: 0.933; Data completeness; 0.998, final R-indices [I > 2 σ(I)]: R1 = 0.061, wR2 = 0.183; largest diff. peak and hole: 0.211and -0.313 eÅ^{-3}.

Crystal data for **3**: $C_{26}H_{28}N_2O_6$, crystal system, space group: monoclinic, P21/n; (no:14); unit cell dimensions: a =9.683(3), b = 25.410(7), c = 10.344(3) Å, α= 90, β= 111.007(5), γ = 90º; volume; 2375.9 (3) Å3, Z=4; calculated density: 1.299 g/cm^3; absorption coefficient: 0.093 mm^{-1}; F(000): 984; θ-range for data collection 1.6-27.5º; refinement method: full matrix least-square on F2;

data/parameters: 3147/313; goodness-of-fit on F2: 1.016; Data completeness; 1.000, final R-indices [I > 2 σ(I)]: R1 = 0.049, wR2 = 0.121; largest diff. peak and hole: 0.145and -0.182 eÅ^{-3}.

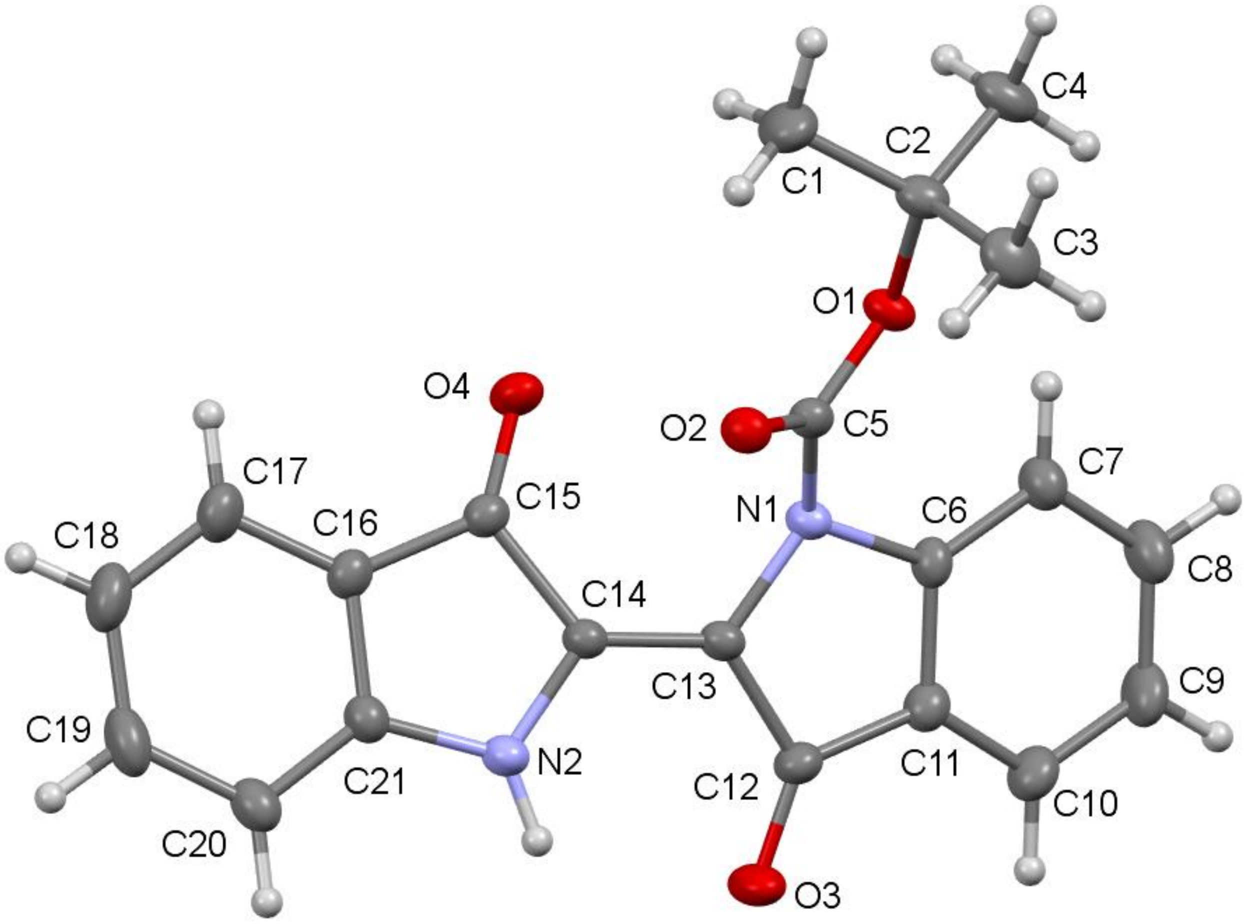

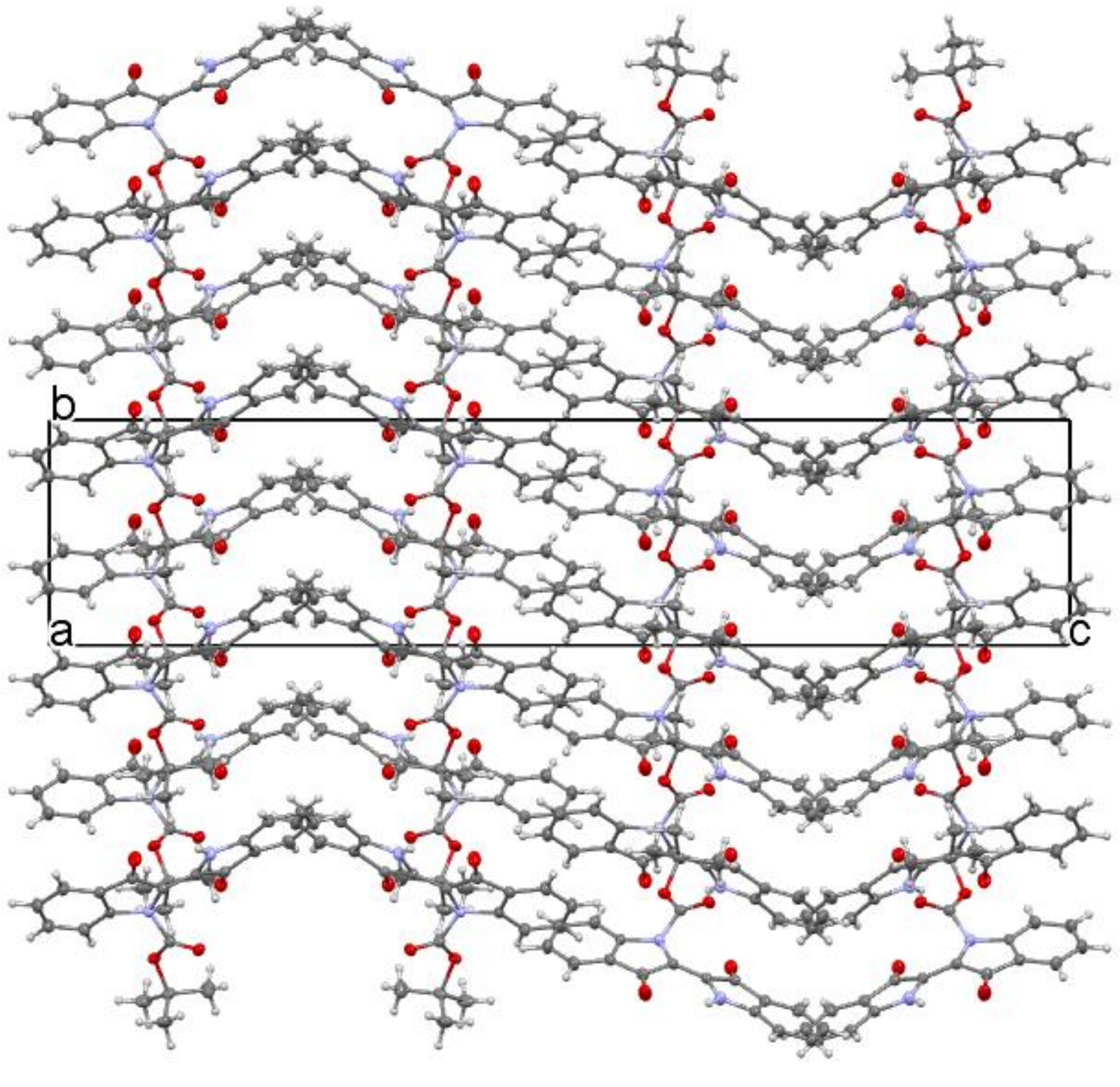
b
a
c

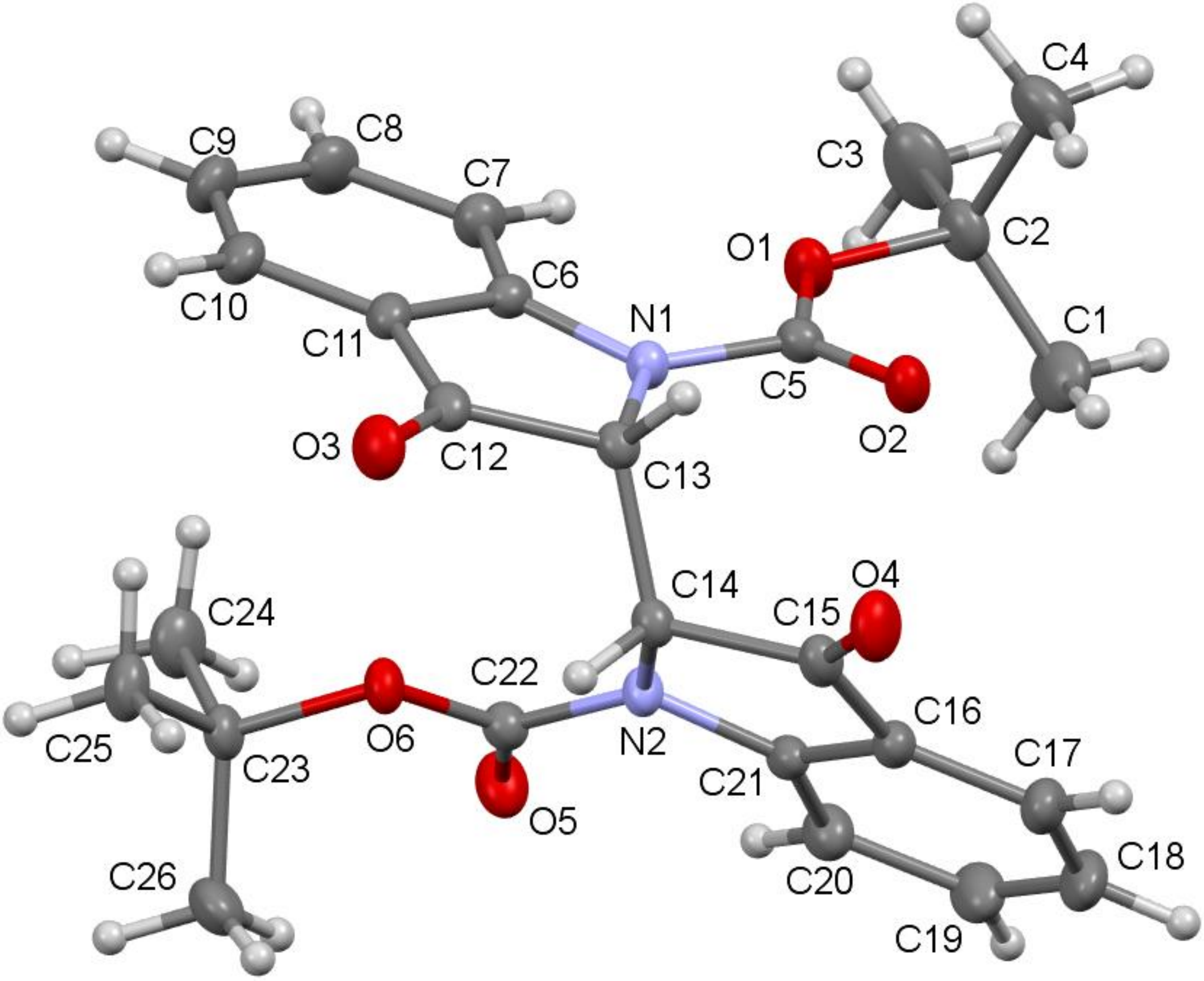

C4
C9
C8
C3
C7
C2
O1
C6
C10
N1
C1
C11
C5
O2
O3
C12
C13
O4
C14
C24
C15
C22
C16
O6
N2
C25
C23
C17
C21
O5
C18
C26
C20
C19

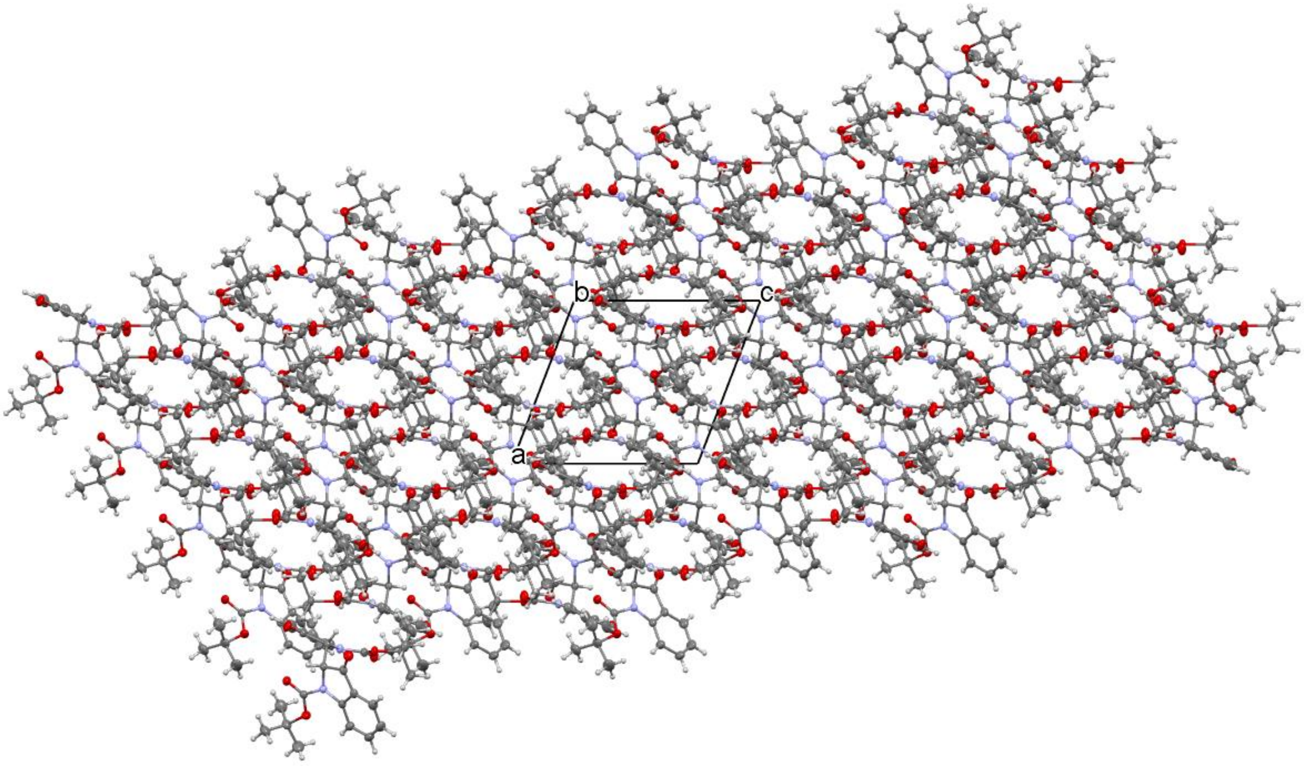

Figure S12. (top) The stacking motif and molecular structure of **2** with the unit cell viewed down along the a-axis. (bottom) The stacking motif and molecular structure of **3** with the unit cell viewed down along the b-axis. Thermal ellipsoids are drawn at the 40 % probability level.

Mass spectra

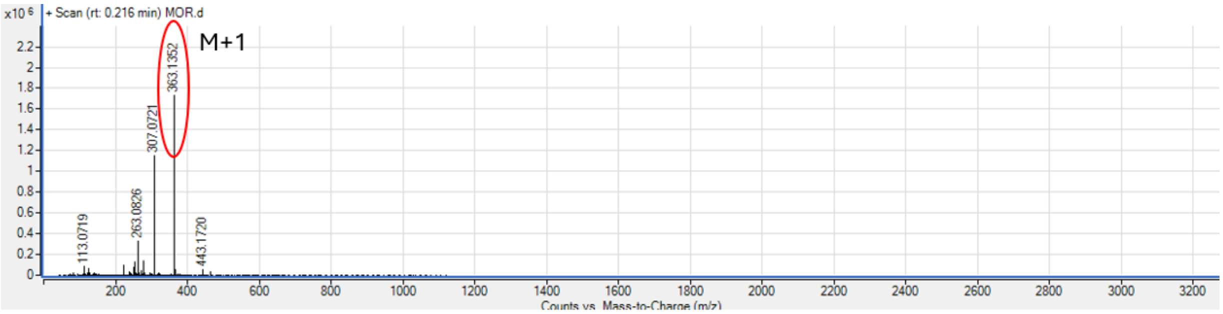

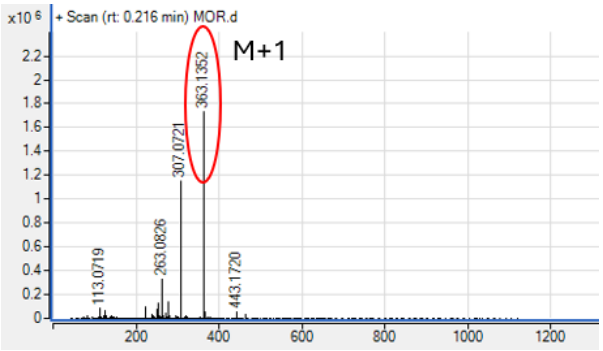

Figure S13. Mass spectrum of **2** (calculated m/z: 363.1267).

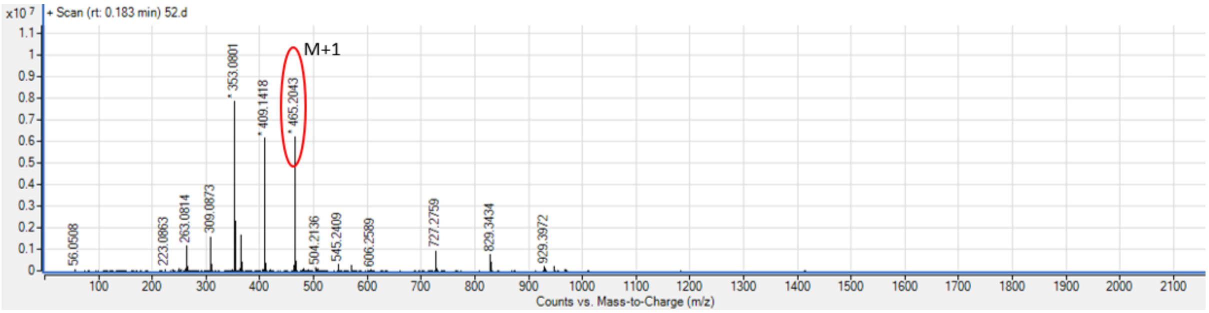

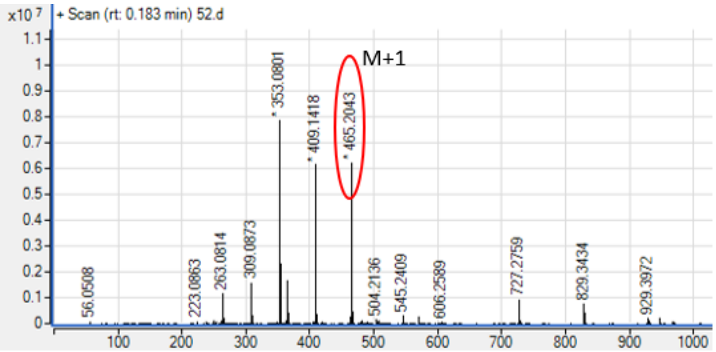

Figure S14. Mass spectrum of **3** (calculated m/z: 464.1947).

IR spectra

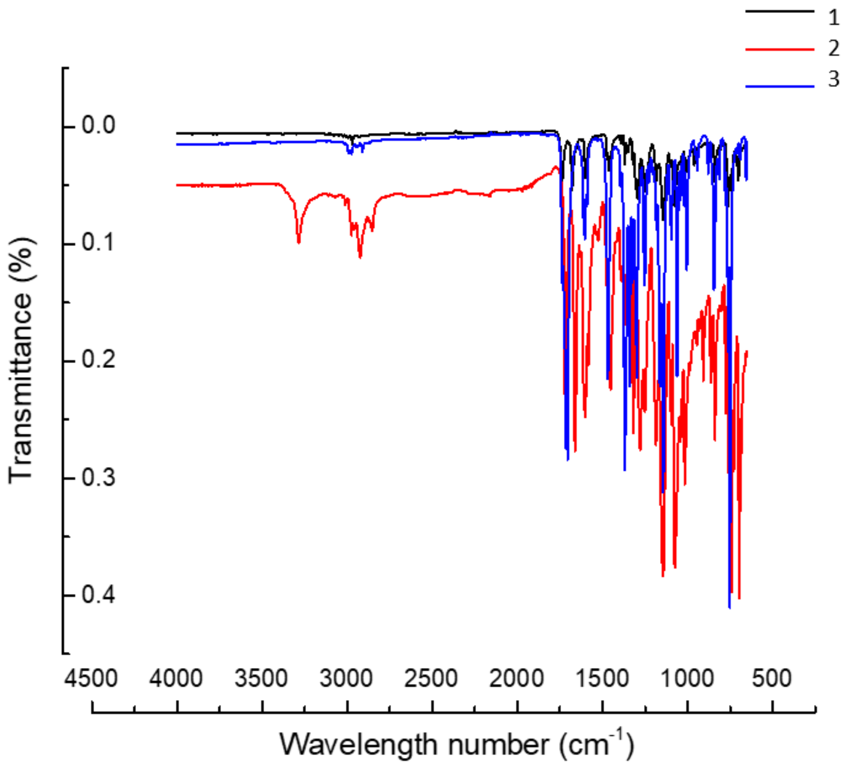

Figure S15. IR spectra of compounds.

NMR spectra

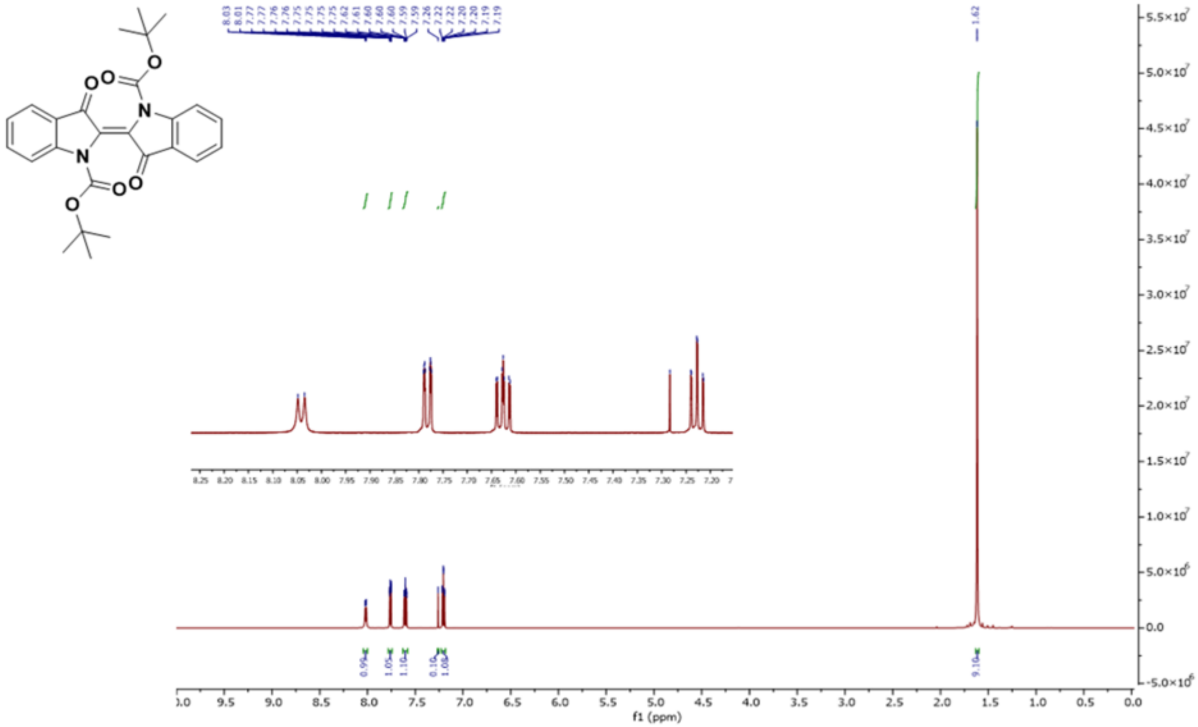

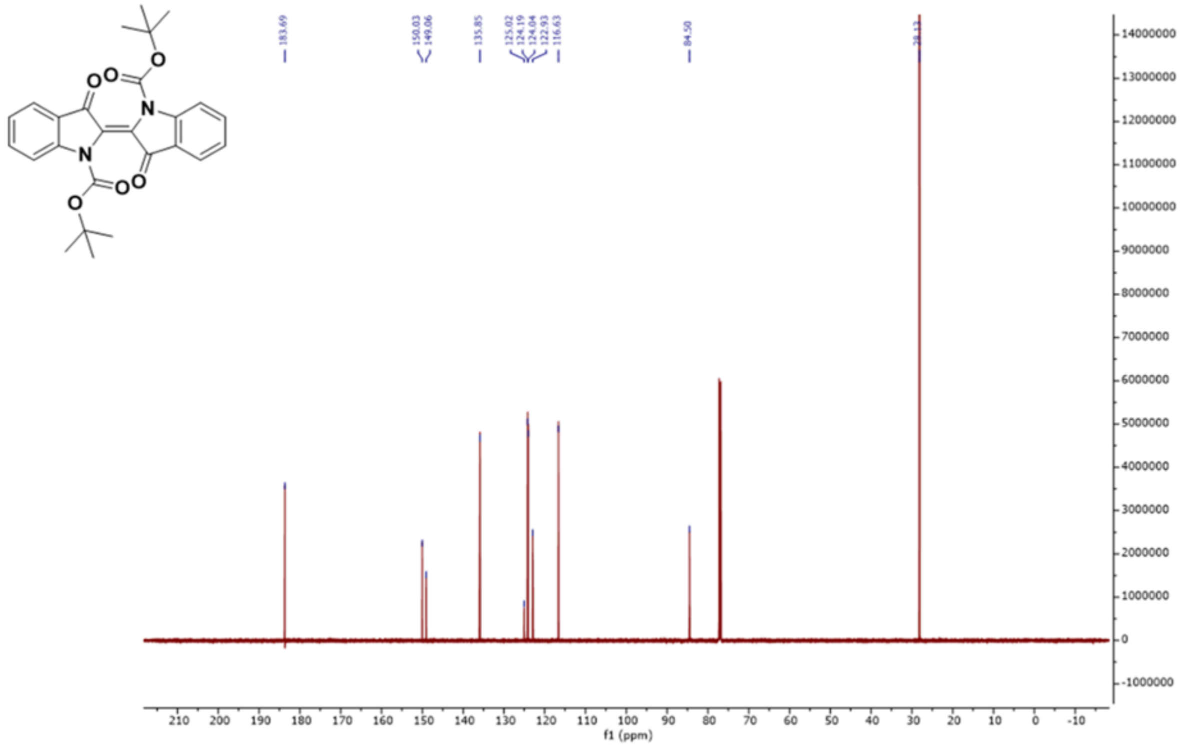

Figure S16. ^{1}H-NMR (up, 600 MHz) and ^{13}C{^{1}H}-NMR (down, 151 MHz) spectra of **1** in $CDCl_3$.

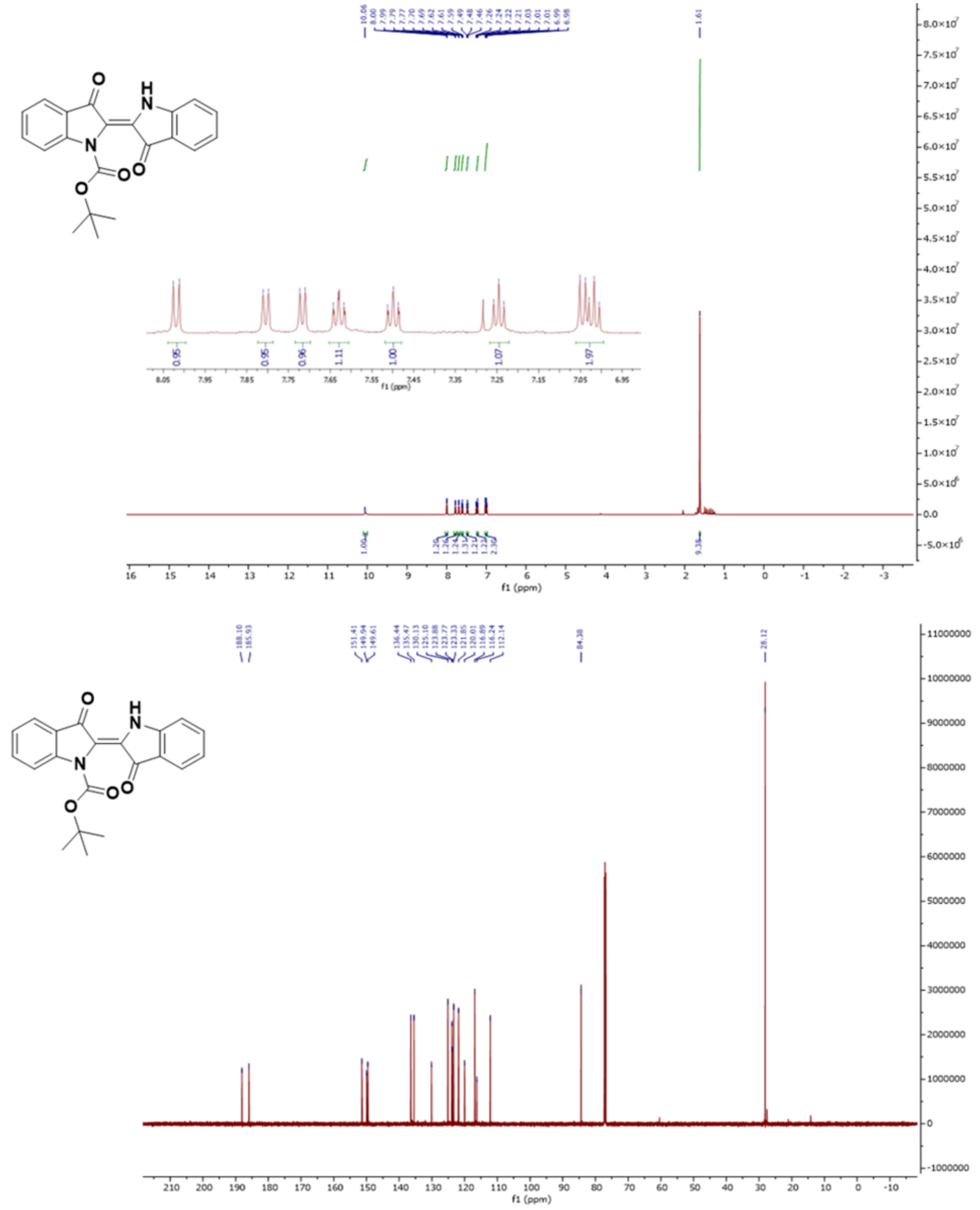

Figure S17. ^{1}H-NMR (up, 600 MHz) and $^{13}C\{^{1}H\}$-NMR (down, 151 MHz) spectra of **2** in $CDCl_3$.

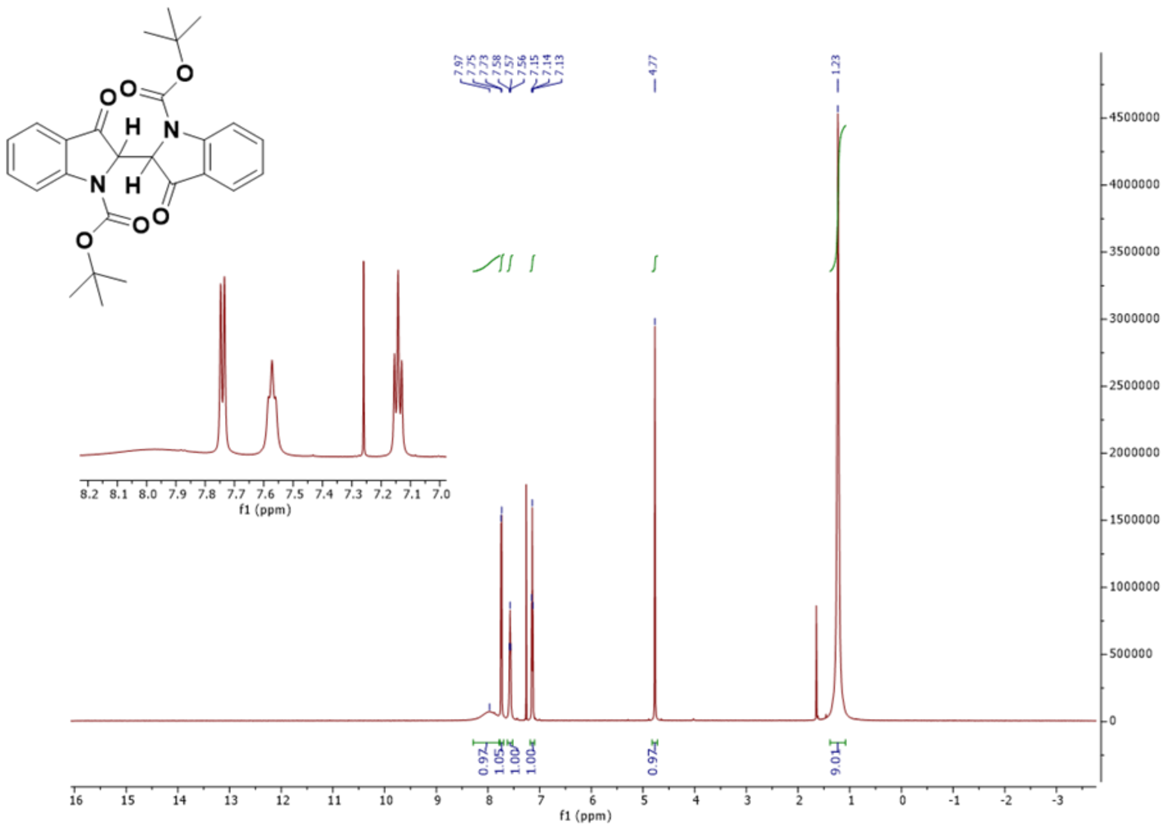

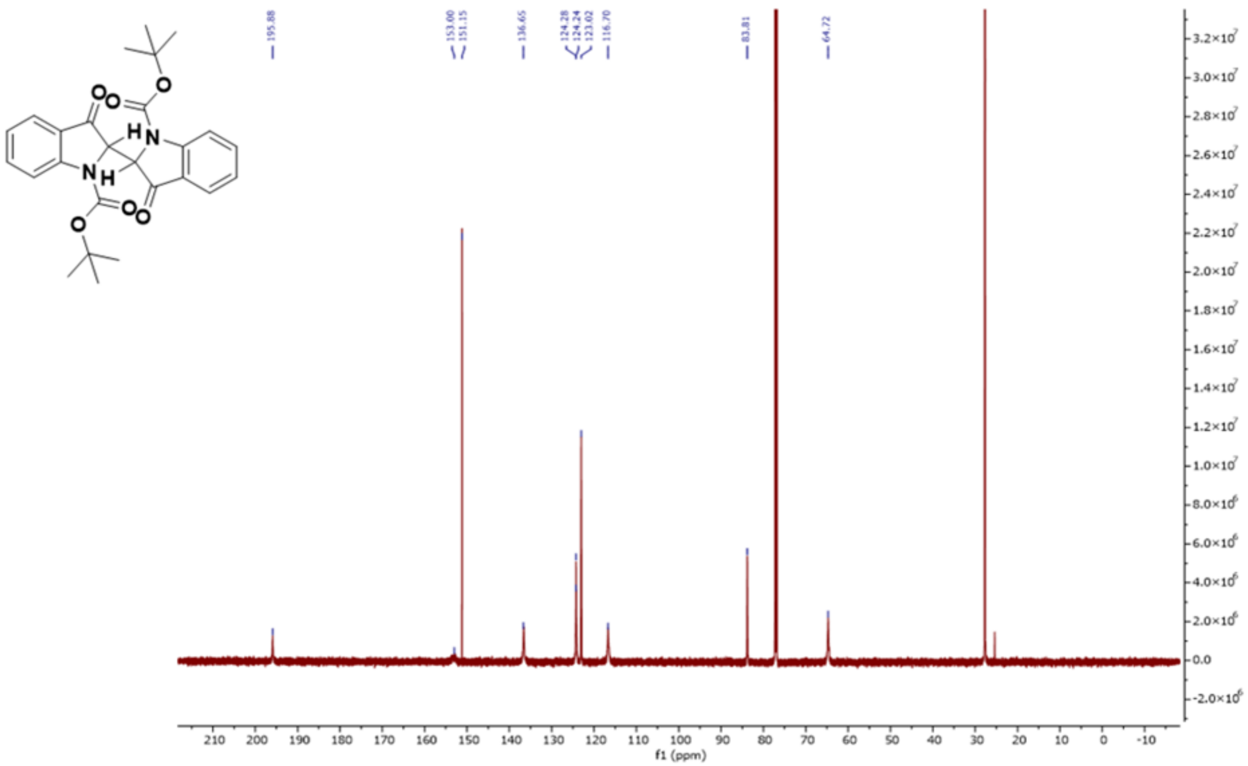

Figure S18. ^{1}H-NMR (up, 600 MHz) and $^{13}C\{^{1}H\}$-NMR (down, 151 MHz) spectra of **3** in $CDCl_3$.

Dyeing studies of textiles

Fabric samples made of 100 % Cotton, 100% Polypropylene (PP), and a blend of 86% Polyethylene terephthalate (PET) and 14 % Elastane were cut into A4-size sheets and sewn together one after the other to form a continuous strip. The dyeing solution was prepared by dissolving 10 g of **1**, 10 g of sodium hydroxide (NaOH) and 20 g of sodium dithionite ($Na_2S_2O_4$) in a 1 L volumetric flask and then making up to a total volume of 1 L with distilled water. The solution was stirred overnight at room temperature to ensure complete dissolution and reaction. The resulting yellow coloration of the solution indicated the completion of the reduction process.

The prepared solution was then transferred to the vessel of the dyeing machine. The sewn fabrics were dyed in pad-batch dyeing machine with 2 bar foulard pressure (squeezed between rollers to remove excess liquid), 2 m/min. passing speed. The fabrics were then exposed to air to allow oxidation. Immediately after oxidation, a visible color change of the fabrics was observed, confirming the success of the dyeing process. After the dyeing procedure was completed, fabrics were washed with water at 70 °C and dried in RAM machine at 120 °C for 3 min.

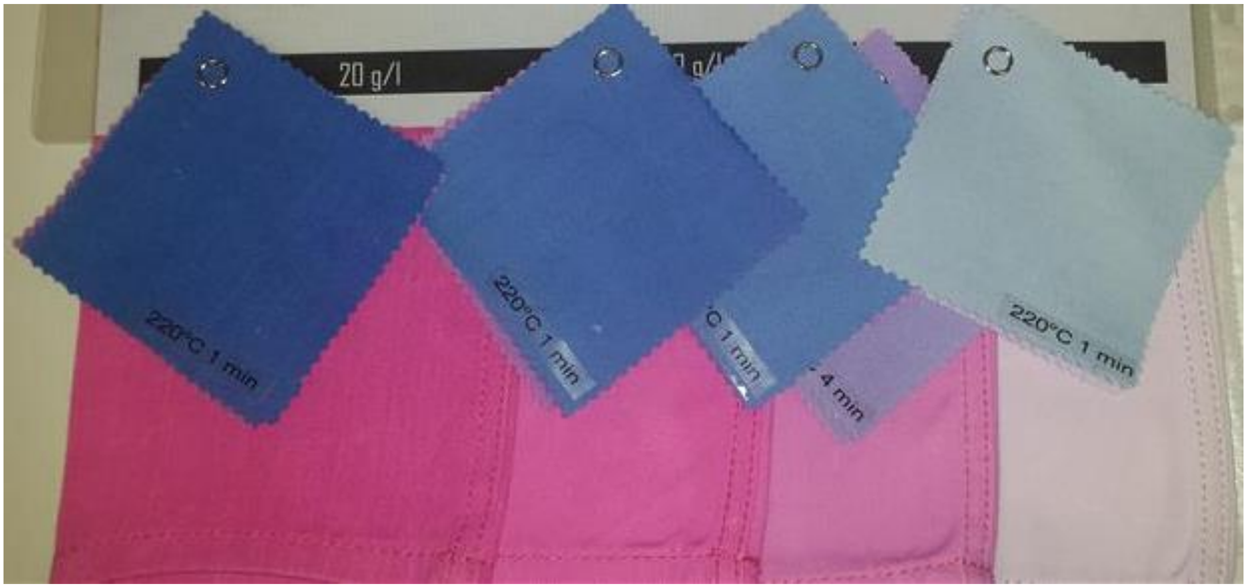

Figure S19. 100 % PP fabrics dyed with various concentrations.

Colors of dyed textiles and color alterations after heat treatment

The color of the dyed textiles changed based on the temperature and the time. The fabrics were kept in stenter at 180 °C for 1 min to 4 min. In addition, higher temperatures of 190 °C, 200 °C, 210 °C and 220 °C were applied for 1 minute. The color of the fabrics changed from magenta to indigo blue.

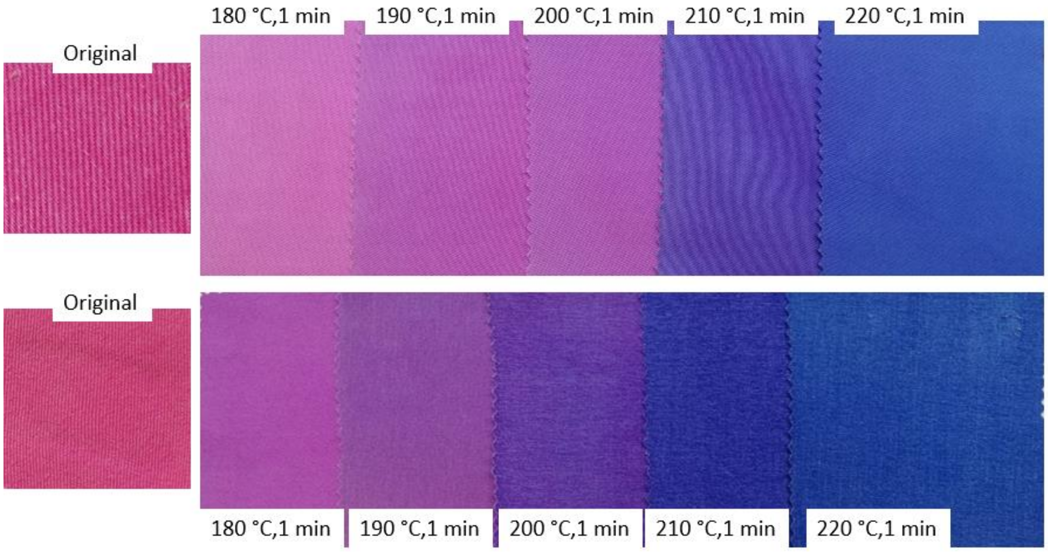

Figure S20. The color alteration of dyed fabrics (100 % PP (up) and 86 % (PET) and 14 % elastane (down) before and after thermal treatment.

Color coordinates of dyed textiles and after thermal treatment

The color coordinates of the dyed textile samples were analyzed before and after thermal treatment and showed in CIELAB color space.

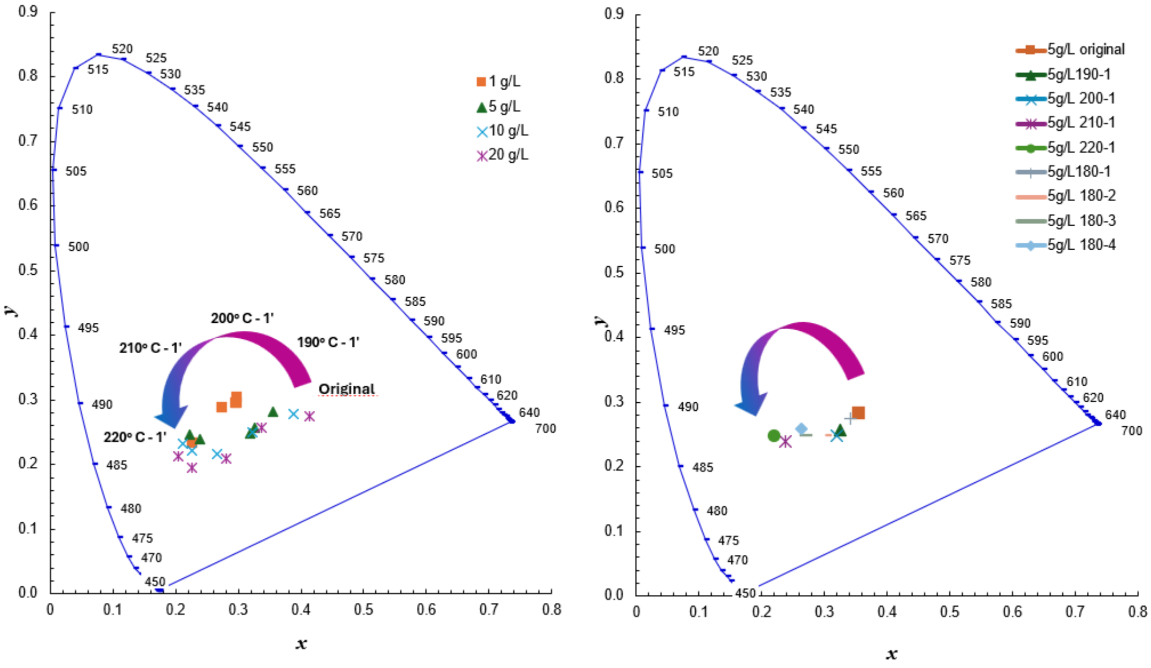

Figure S21. The color coordinates of the dyed textile samples (86 % PET-14 % elastane) before and after thermal treatment were plotted on the CIE x, y chromaticity diagram.

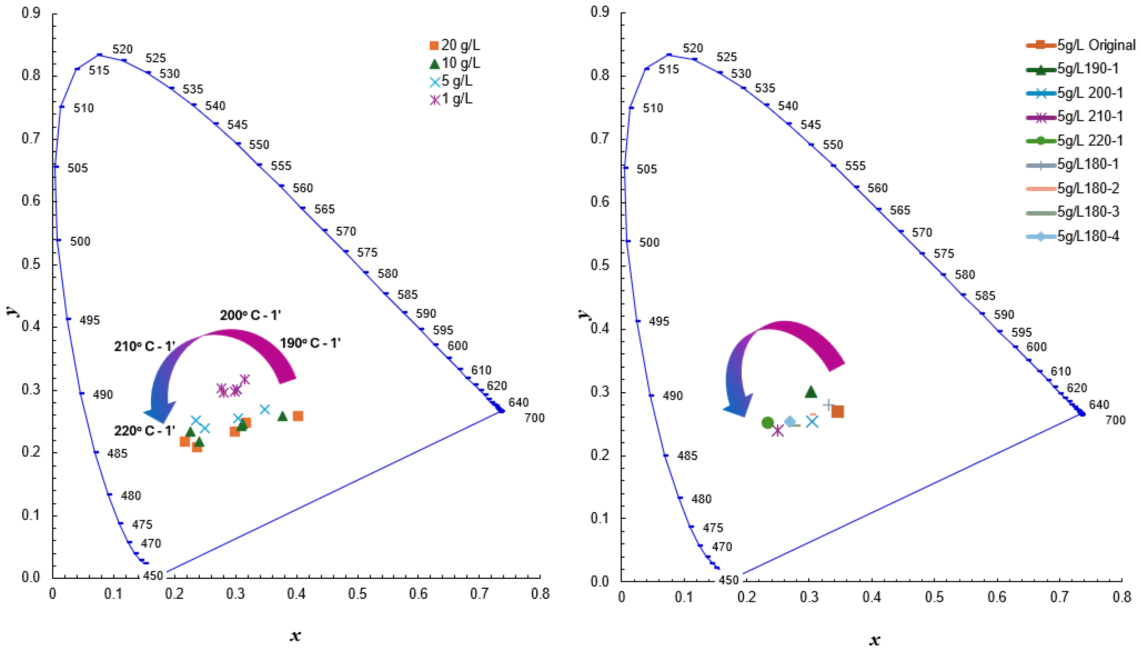

Figure S22. The color coordinates of the dyed textile samples (100 % PP) before and after thermal treatment were plotted on the CIE x, y chromaticity diagram.

Color fastness of dyed fabrics

Color fastness tests were carried out for washing, water, perspiration (acid and alkaline), and crocking using the following standards: TS EN ISO 105-C06:2010 (washing), TS EN ISO 105-E01:2013 (water), TS EN ISO 105-E04:2013 (perspiration-acid and alkaline), and EN ISO 105-X12:2016 (crocking) respectively.

Table S1. Color fastness analysis of dyed textile samples (5 g/L dye concentration)

Colorfastness to[a]		**86 % PET-14 % Elastane**	**100 % PP**
Perspiration (alkaline)	Acetate	4	4
	Cotton	4	4
	Nylon	4	4
	PET	4	4
	Acrylic	4	4
	Wool	4	4
Perspiration (acidic)	Acetate	4	4.5
	Cotton	4	4
	Nylon	4	4
	PET	4	4
	Acrylic	4	4
	Wool	4	4
Water	Acetate	4.5	4
	Cotton	4	4
	Nylon	4	4
	PET	4	4
	Acrylic	4	4
	Wool	4	4
Washing	Acetate	4	4

	Cotton	4	4
	Nylon	4	4
	PET	4	4
	Acrylic	4.5	4.5
	Wool	3.5	3.5
Dry crocking[b]	-	3	3
Wet crocking[c]	-	3.5	3.5
Light	-	1	1
Dry cleaning	-	1	1
Oxidative bleaching	-	1	1

[a] Rate for all fastness: 1-2 (not acceptable); 3-5 (acceptable), [b] Rate for dry crocking fastness: 1-2 (not acceptable); 3-5 (acceptable), [c] Rate for wet crocking fastness: 1-1.5 (not acceptable); 2-5 (acceptable).

Cartesian coordinates of computed compounds in Figures 4 and 9

3-diketo							
0 1							
O	1.94216805	1.23847302	0.88465166	C	-2.55499095	1.01559165	1.25051182
O	0.84384553	2.67650260	-0.49729942	C	-1.89418726	-0.06326022	1.98991036
O	-2.38182682	-0.90840786	2.70684265	C	-0.38908437	0.06781895	1.69862351
O	-0.06814057	-2.08825716	-2.18456436	H	0.08101022	0.40503244	2.63119797
O	-1.58146591	-1.85920181	-0.49623217	C	0.29520670	-1.28320726	1.35479140
O	1.84073214	-1.21773054	3.23731136	H	-0.35472973	-2.06541746	1.76114110
N	-0.31780478	1.13854728	0.69020756	C	1.66859006	-1.41070735	2.05522852
N	0.59887801	-1.54606004	-0.06028620	C	2.62963740	-1.79667569	1.02266142
C	2.98265512	2.41153137	-1.70397991	C	3.99257384	-2.05620009	1.13952112
H	3.40014869	1.66805316	-1.02747753	H	4.46577486	-2.00338878	2.11302811
H	3.80487372	2.96901888	-2.16075198	C	4.70706412	-2.37623314	-0.00809719
H	2.43643825	1.90037770	-2.50053909	H	5.76850034	-2.58649538	0.04709890
C	2.06546872	3.38888851	-0.96791661	C	4.04865823	-2.42411360	-1.24414651
C	2.75598721	4.07195386	0.21338817	H	4.61445138	-2.67385067	-2.13530134
H	2.05233790	4.71467371	0.74868578	C	2.68551098	-2.15981689	-1.37552960
H	3.56834014	4.70006187	-0.16200875	H	2.19411656	-2.20168932	-2.33439843
H	3.17203917	3.34436588	0.90809644	C	1.97467554	-1.84307430	-0.21548828
C	1.48645549	4.42345894	-1.93263281	C	-0.34669408	-1.85456836	-1.02762999
H	0.94977180	3.93488059	-2.74903770	C	-2.75572602	-2.29028160	-1.31048552
H	2.29448510	5.02020916	-2.36184203	C	-2.99072119	-1.29035675	-2.44355925
H	0.80078797	5.09789532	-1.41450017	H	-3.08840997	-0.27780904	-2.04476774
C	0.92642714	1.67511634	0.38680316	H	-3.92291568	-1.54296745	-2.95637763
C	-1.59971154	1.68815582	0.47116262	H	-2.17812782	-1.31191358	-3.16810860
C	-1.99957533	2.73693566	-0.36308934	C	-3.90017985	-2.25507924	-0.29980926
H	-1.28946751	3.26173672	-0.98015064	H	-3.68963899	-2.90658032	0.55008737
C	-3.34881029	3.08589142	-0.37088487	H	-4.81981848	-2.59600035	-0.78164014
H	-3.66767215	3.89997972	-1.01279459	H	-4.06179146	-1.24398506	0.07398593
C	-4.30024165	2.42543455	0.41829779	C	-2.53940038	-3.71770840	-1.81901438
H	-5.33805751	2.73434223	0.38658999	H	-1.73579859	-3.76977949	-2.55097041
C	-3.90117425	1.37574050	1.23495565	H	-3.46230489	-4.06942601	-2.28780547
H	-4.60518590	0.83690121	1.85862943	H	-2.30964978	-4.38807039	-0.98674826

3-enol							
0 1				H	-2.17501317	-4.32347374	1.43631410
O	-0.34200393	2.01805870	1.60513780	C	-1.69561456	-2.24698944	1.17089922
O	-2.28098136	1.82635228	0.42438807	C	-0.33812077	-2.14661723	1.69066451
O	0.37560780	-3.00306363	2.18375565	C	0.07463156	-0.67446681	1.56071449
O	1.28700649	1.02919978	-2.34903716	H	0.16482507	-0.28999607	2.58693667
O	0.30732119	-0.87290707	-1.57967659	C	1.43192442	-0.48970209	0.92441316
O	2.69520313	-1.37299853	2.80635677	C	2.58155942	-0.72933025	1.61738514
N	-1.08519917	-0.02716529	0.93237939	C	3.67403331	-0.07864687	0.94700204
N	1.74143990	0.26701171	-0.23496174	C	5.03948192	0.02419579	1.22960235
C	-1.46237955	4.02273211	-0.35688176	H	5.45091022	-0.45482629	2.10983534
H	-0.52541527	3.99805889	0.19652593	C	5.84107324	0.74890438	0.35728634
H	-1.75661353	5.06612509	-0.49902107	H	6.90177929	0.84944451	0.55695814
H	-1.30204864	3.57821387	-1.34197776	C	5.29233368	1.35360286	-0.78455837
C	-2.57650612	3.28855881	0.39002861	H	5.93691105	1.91720557	-1.44965294
C	-2.77735095	3.81025024	1.81341727	C	3.93455826	1.26055937	-1.08264097
H	-3.53738361	3.22192134	2.33414896	H	3.51350499	1.73618461	-1.95590468
H	-3.12448947	4.84597826	1.76855727	C	3.13030170	0.55088095	-0.19316922
H	-1.85063131	3.78001945	2.38396258	C	1.09481306	0.20765531	-1.48305201
C	-3.88511463	3.33656157	-0.39805207	C	-0.33170528	-1.22585627	-2.88132930
H	-3.75212641	2.91446175	-1.39664637	C	-1.34019075	-0.14861071	-3.28327811
H	-4.21310760	4.37297351	-0.50478271	H	-2.06211454	0.01756717	-2.48065308
H	-4.67175621	2.78077229	0.11776483	H	-1.88864371	-0.48534080	-4.16741861
C	-1.18410241	1.36270881	1.03689214	H	-0.84454804	0.79171050	-3.51899774
C	-2.11109379	-0.97155502	0.74103266	C	-1.03272014	-2.54560858	-2.56475069
C	-3.40014527	-0.80390723	0.22391055	H	-0.31357149	-3.28861338	-2.21392053
H	-3.74510227	0.16123156	-0.10907667	H	-1.51217588	-2.92921854	-3.46861861
C	-4.22602399	-1.92345354	0.15409267	H	-1.79839767	-2.41277274	-1.79995976
H	-5.22680070	-1.80230631	-0.24624126	C	0.75007841	-1.44138681	-3.94270848
C	-3.81080255	-3.19460918	0.57812512	H	1.25962129	-0.51343086	-4.19520346
H	-4.48765773	-4.03693701	0.50369843	H	0.28414788	-1.84021151	-4.84777120
C	-2.53339910	-3.36000063	1.09310731	H	1.48648597	-2.16956902	-3.59364037
				H	2.02138526	-2.07644641	2.83654891

3-di-enol							
0 1							
O	-0.08831762	1.21968216	1.40232766	C	-1.95300991	-2.58060388	-0.19363782
O	-2.34347940	1.30064380	1.16581863	C	-0.52347182	-2.56185594	-0.18362936
O	0.31780257	-3.62064131	-0.42296570	C	-0.07036707	-1.31170349	0.12815985
O	2.21902770	2.00910547	-1.63006889	C	1.32733504	-0.95196076	0.27622781
O	0.44083096	0.60754298	-1.82895959	C	2.23818635	-1.66935827	1.01307882
O	2.04732424	-2.82645060	1.69170544	C	3.48378522	-0.95994635	1.03962541
N	-1.21779970	-0.49452391	0.37497276	C	4.70504841	-1.22669714	1.66655075
N	1.99957062	0.19230048	-0.25161056	H	4.82469591	-2.12163790	2.26511709
C	-1.79202297	3.71039364	1.28763119	C	5.74281862	-0.32031228	1.50057036
H	-0.71325601	3.56720972	1.30545601	H	6.69908901	-0.49951758	1.97864900
H	-2.03052949	4.63660097	1.81753045	C	5.56478302	0.83088467	0.71660871
H	-2.12057369	3.82242102	0.25148884	H	6.38753712	1.52780591	0.60215873
C	-2.52476382	2.54886770	1.96003823	C	4.35681690	1.11097343	0.08264109
C	-2.06323070	2.31769479	3.39994425	H	4.22685125	1.99878943	-0.51565068
H	-2.56062647	1.44232849	3.82526730	C	3.31137350	0.20273970	0.25874670
H	-2.33362275	3.18716860	4.00498362	C	1.58787112	1.03641022	-1.28136803
H	-0.98518473	2.17740331	3.45807838	C	-0.12397530	1.26353085	-3.03508001
C	-4.03832726	2.75474942	1.90139971	C	-0.51131748	2.70934050	-2.71932076
H	-4.37584145	2.86206038	0.86795052	H	-1.18810479	2.74332737	-1.86249519
H	-4.30801555	3.66257948	2.44584242	H	-1.03353087	3.13659379	-3.57994409
H	-4.56396467	1.91263061	2.35703567	H	0.36468868	3.31838741	-2.50330095
C	-1.13171965	0.74533548	1.02814763	C	-1.36470680	0.41767334	-3.32088765
C	-2.37305639	-1.27035975	0.14141642	H	-1.08853142	-0.62346622	-3.49997094
C	-3.73194261	-0.95484250	0.17875078	H	-1.87393430	0.79894758	-4.20932148
H	-4.06690007	0.03909967	0.42407931	H	-2.06188732	0.44992256	-2.48169792
C	-4.64963386	-1.96197115	-0.11280411	C	0.87318183	1.16532348	-4.19212411
H	-5.70766781	-1.72786183	-0.08701588	H	1.76264291	1.76490862	-4.00608045
C	-4.23990687	-3.26236773	-0.43804862	H	0.39558956	1.52376588	-5.10807299
H	-4.98167602	-4.02150872	-0.65668768	H	1.17109060	0.12574425	-4.35068662
C	-2.88953970	-3.58050790	-0.48079251	H	1.42231580	-3.38239264	1.20292058
H	-2.57410828	-4.58938688	-0.72801433	H	-0.16569608	-4.34399981	-0.83508306

3a-TS1							
-1 1				C	-2.61242094	0.83702511	1.11756308
O	1.85931793	1.27835212	0.56732863	C	-1.86608234	-0.19242457	1.84384368
O	0.61565263	2.62415716	-0.78228671	C	-0.41900496	0.04278570	1.56476685
O	-2.31326484	-1.05898608	2.59373050	H	-0.06129703	0.59834295	2.59738839
O	0.08497366	-2.12345738	-2.23331007	C	0.38737405	-1.24984081	1.31310065
O	-1.43671748	-2.07001969	-0.54122489	H	-0.20874757	-2.06130371	1.74610055
O	1.94696124	-1.15197313	3.19540992	C	1.76474339	-1.30405751	2.00074384
N	-0.40794359	1.07194178	0.50217511	C	2.75029781	-1.62850309	0.97367419
N	0.70805966	-1.56590666	-0.10165902	C	4.12952940	-1.79012135	1.09312677
C	2.68482117	2.45588800	-2.12177210	H	4.60449353	-1.67960887	2.06119656
H	3.18739587	1.75047220	-1.46225381	C	4.86577671	-2.09060673	-0.04614980
H	3.44218641	3.05040453	-2.63907509	H	5.93953614	-2.22149960	0.01153746
H	2.11818423	1.90060830	-2.87333623	C	4.20946482	-2.22219247	-1.27838701
C	1.76228853	3.39410146	-1.34313394	H	4.79020492	-2.45453373	-2.16435563
C	2.48678270	4.14323974	-0.22430558	C	2.83033251	-2.06164848	-1.41099457
H	1.78103840	4.74997761	0.34841598	H	2.34852265	-2.16401035	-2.36960341
H	3.22789698	4.81319885	-0.66769553	C	2.09523519	-1.76021239	-0.25957017
H	2.99722082	3.45893659	0.45112937	C	-0.21360565	-1.93726020	-1.06742936
C	1.06612414	4.37443469	-2.28689537	C	-2.59105376	-2.52787266	-1.37589984
H	0.50635121	3.83984679	-3.05761131	C	-2.90335457	-1.47420134	-2.43843880
H	1.81230677	5.00363548	-2.77688354	H	-3.06458518	-0.49889262	-1.97359629
H	0.37868446	5.02115822	-1.73712837	H	-3.82037295	-1.75674860	-2.96183779
C	0.78923926	1.65383747	0.12405034	H	-2.09967371	-1.39311877	-3.16896086
C	-1.71554737	1.55984020	0.30981525	C	-3.72391452	-2.63081109	-0.35710023
C	-2.19657461	2.58939600	-0.51003258	H	-3.47780256	-3.35395513	0.42314815
H	-1.53632214	3.15893428	-1.14219183	H	-4.63345591	-2.96356486	-0.86229085
C	-3.56382806	2.85644692	-0.49228043	H	-3.91917626	-1.66563753	0.11011839
H	-3.94363670	3.64917617	-1.12754206	C	-2.29312292	-3.90427798	-1.97422584
C	-4.45848756	2.13660927	0.31397035	H	-1.51437119	-3.85865647	-2.73267993
H	-5.51424638	2.37950466	0.30045772	H	-3.20609491	-4.29121369	-2.43406729
C	-3.97943405	1.11620097	1.12478257	H	-1.98878221	-4.60286653	-1.19063042
H	-4.64333725	0.54081491	1.75997553	O	0.20837118	1.41080832	3.82474322
				H	0.96124244	0.92265334	4.17660279

3b			
-1 1			
O	0.93848506	1.85400082	0.42572069
O	-0.85029149	2.69875962	-0.69831656
O	-1.66796958	-2.27864362	2.22192436
O	1.71151221	-1.63747934	-2.42630466
O	-0.02969166	-2.33979118	-1.14023231
O	1.61639219	-0.06707211	3.20038438
N	-1.04785822	0.72509408	0.37919360
N	1.54061016	-1.02586168	-0.22446725
C	1.00102251	3.64755868	-2.02967184
H	1.79793293	3.14416726	-1.48516961
H	1.39363079	4.58124202	-2.44065247
H	0.68496153	3.01647190	-2.86406446
C	-0.18709711	3.96345391	-1.12004025
C	0.20977916	4.78387669	0.10808608
H	-0.65187521	4.93012410	0.76427470
H	0.55763047	5.76725952	-0.21832503
H	1.00793066	4.30281726	0.67095178
C	-1.29405077	4.66741508	-1.90494084
H	-1.61994393	4.05487140	-2.74852218
H	-0.92083845	5.61778780	-2.29269536
H	-2.15548389	4.87175634	-1.26526711
C	-0.22328762	1.79008588	0.06220662
C	-2.46158477	0.71876744	0.42266160
C	-3.40809696	1.60545674	-0.09669001
H	-3.11416907	2.47741039	-0.65829628
C	-4.75805176	1.32525367	0.12090563
H	-5.50355214	2.00112958	-0.28363900
C	-5.16770967	0.19289554	0.84040219
H	-6.22516538	0.00327004	0.98824430
C	-4.21991725	-0.68252482	1.36236327
H	-4.51788448	-1.56093483	1.92432455
C	-2.86425474	-0.41798645	1.15600729
C	-1.66963551	-1.16485935	1.56579743
C	-0.58632137	-0.44654173	1.07795786
H	-0.79301501	-2.12364256	3.68954911
C	0.80197851	-0.97957143	1.07927103
H	0.69195281	-2.00726532	1.45362453
C	1.82395054	-0.31205190	2.01984442
C	3.05428263	-0.12177718	1.26628664
C	4.27868343	0.39458196	1.69100763
H	4.40253707	0.71195675	2.72003059
C	5.31708029	0.48722314	0.77419047
H	6.28097151	0.88164957	1.07208741
C	5.11062748	0.06563655	-0.54826134
H	5.92547399	0.14197932	-1.25993475
C	3.89182576	-0.44816763	-0.98726218
H	3.75610447	-0.76011248	-2.00997312
C	2.84693678	-0.54137975	-0.05890944
C	1.10787668	-1.67694332	-1.36740814
C	-0.74460447	-3.06426001	-2.22850149
C	-1.16008054	-2.08127193	-3.32407634
H	-1.73189942	-1.25362904	-2.89743861
H	-1.79998002	-2.59977457	-4.04265511
H	-0.29698602	-1.68209119	-3.85449142
C	-1.97183553	-3.61713341	-1.50573001
H	-1.67426855	-4.28503307	-0.69460588
H	-2.58786292	-4.18027196	-2.21030751
H	-2.57118137	-2.80714540	-1.08629160
C	0.12893455	-4.20354482	-2.75635103
H	1.00253366	-3.82742894	-3.28606848
H	-0.46091294	-4.81431012	-3.44471873
H	0.45816376	-4.84351536	-1.93380865
O	-0.25439985	-1.94241326	4.50299961
H	0.36493505	-1.25740206	4.20647838

3c-TS2							
-1 1				H	2.83962345	-3.16006622	-2.93518414
O	0.11521996	1.90892177	0.50239406	C	2.15279386	-1.47952637	-1.76978528
O	2.38757922	1.92583606	0.63838043	C	0.71532930	-1.46305191	-1.84205695
O	-0.18670468	-2.23021683	-2.44775676	C	0.25617708	-0.41233692	-1.10148860
O	-2.68840521	-1.14496975	2.07822177	C	-1.20277845	-0.26857699	-1.17236610
O	-0.90463196	-2.01102821	0.96214349	H	-1.11830497	-1.47765826	-1.96467283
O	-1.33370372	1.20559669	-3.09706201	C	-1.80333059	0.74397169	-2.04267212
N	1.35582376	0.30041573	-0.54052647	C	-3.09604082	1.10182995	-1.44104940
N	-2.08357593	-0.34887970	0.01603763	C	-4.09171154	1.96083716	-1.90661766
C	1.80149583	2.92253926	2.82321719	H	-3.97076309	2.45691526	-2.86331007
H	0.72323999	2.82295397	2.71068170	C	-5.22600983	2.15878103	-1.12856584
H	2.00681662	3.77343733	3.47777510	H	-6.01610618	2.81901174	-1.46689736
H	2.19501470	2.02313867	3.30342818	C	-5.34769218	1.49659418	0.10350605
C	2.48879947	3.15310067	1.47679839	H	-6.23542650	1.65707582	0.70587185
C	1.91731679	4.35115677	0.71751682	C	-4.36086075	0.63971417	0.58552284
H	2.39374966	4.44503628	-0.26154127	H	-4.47027908	0.14603056	1.53764077
H	2.12404680	5.26278568	1.28393024	C	-3.21429569	0.44969147	-0.20059605
H	0.84062790	4.26334937	0.58205140	C	-1.94455223	-1.17924943	1.10840400
C	3.99932069	3.29991095	1.65935911	C	-0.51728769	-2.97404016	2.02414312
H	4.41612825	2.42538964	2.16394389	C	-0.12263149	-2.22261649	3.29705518
H	4.21113464	4.18183778	2.26777165	H	0.64551458	-1.47711505	3.07666175
H	4.49689902	3.41953712	0.69441865	H	0.29170177	-2.93285845	4.01730279
C	1.20551095	1.43954048	0.23784619	H	-0.97897729	-1.72696388	3.75146660
C	2.53195851	-0.37393369	-0.95127532	C	0.69840624	-3.66975950	1.41177402
C	3.87786247	-0.12362089	-0.68072874	H	0.42682399	-4.17223726	0.48110358
H	4.17824833	0.70733536	-0.06301181	H	1.08305343	-4.41688203	2.10978340
C	4.83391340	-0.97768557	-1.23271195	H	1.49145681	-2.95026779	1.19968760
H	5.88224804	-0.78939450	-1.02941205	C	-1.64640243	-3.97967780	2.25959434
C	4.46985478	-2.06618194	-2.03971836	H	-2.51809605	-3.50576106	2.70744044
H	5.23895906	-2.70951697	-2.45238474	H	-1.29082828	-4.76495628	2.93184778
C	3.13043219	-2.32162282	-2.31222182	H	-1.94053625	-4.44875053	1.31716280

3d			
-1 1			
O	-0.21143915	-1.05389794	-1.60047090
O	-2.41322280	-1.18110916	-1.06462131
O	0.40067402	3.67739915	0.48327830
O	2.10339262	-2.31822945	1.17788970
O	0.49277145	-0.80235328	1.70868684
O	2.18003402	3.03355403	-1.18350797
N	-1.19736287	0.55894821	-0.29566576
N	1.98322715	-0.29918732	0.09645048
C	-1.93785309	-3.55810835	-1.58494326
H	-0.87139904	-3.41631654	-1.74990136
H	-2.27647540	-4.40282164	-2.19058134
H	-2.10703168	-3.80735381	-0.53501744
C	-2.73795353	-2.31616369	-1.97793833
C	-2.49794936	-1.89664602	-3.42873141
H	-3.03504311	-0.97187102	-3.65352092
H	-2.87692736	-2.67839737	-4.09174711
H	-1.43854811	-1.75283229	-3.63600662
C	-4.22840793	-2.53023964	-1.71557341
H	-4.40665575	-2.76255684	-0.66326106
H	-4.58946219	-3.36634316	-2.31839432
H	-4.80329787	-1.64078474	-1.98213454
C	-1.19918936	-0.61638931	-1.04799470
C	-2.32142148	1.37121461	0.00047105
C	-3.68669299	1.09058151	0.03926578
H	-4.06405777	0.10525890	-0.17976292
C	-4.56363736	2.12452998	0.37772355
H	-5.62728134	1.91731442	0.41367946
C	-4.09770202	3.41331226	0.67564060
H	-4.80495330	4.19287716	0.93642135
C	-2.73600052	3.69430414	0.64268348
H	-2.36367172	4.68563177	0.87428683
C	-1.84319151	2.66950979	0.30434862
C	-0.41215955	2.62850981	0.22750025
C	-0.01301210	1.34840888	-0.09220028
C	1.36979204	0.96016359	-0.23225074
H	1.18906036	3.57339475	-0.17372602
C	2.33745385	1.80529546	-0.78400952
C	3.56768706	1.02831023	-0.87597151
C	4.82794579	1.35141296	-1.38668971
H	5.01189402	2.34112517	-1.79010678
C	5.83215131	0.38740144	-1.36418796
H	6.81547943	0.61783729	-1.75920033
C	5.57824820	-0.88582534	-0.83221665
H	6.36827930	-1.62876573	-0.82288487
C	4.32513042	-1.22713707	-0.31780509
H	4.13868475	-2.21002595	0.08614823
C	3.32025809	-0.25901955	-0.35215063
C	1.55298889	-1.24144916	1.01984762
C	-0.07934771	-1.56900358	2.84872568
C	-0.64268488	-2.90506305	2.36468471
H	-1.36930397	-2.74567916	1.56544735
H	-1.15672488	-3.39604412	3.19519847
H	0.14503392	-3.56454042	2.00440477
C	-1.20255691	-0.65120621	3.33066515
H	-0.80289554	0.31391928	3.64943507
H	-1.71268102	-1.11169222	4.17978473
H	-1.93184642	-0.48159975	2.53650202
C	0.98027118	-1.73900904	3.93919474
H	1.78344475	-2.40083884	3.61906071
H	0.51043843	-2.16667206	4.82858173
H	1.40421096	-0.76962414	4.21323637

3e							
-1 1				C	-1.64122013	2.64390459	0.28494419
O	-0.62911449	-1.63557677	-0.84970522	C	-0.22420707	2.41750282	0.15725042
O	-2.76515521	-0.99917990	-1.28040639	C	-0.02837083	1.13077718	-0.27214224
O	0.67804182	3.37647124	0.42291179	C	1.25941343	0.48294581	-0.66167022
O	2.02650382	-2.03014668	1.90534714	H	1.52058967	3.17112409	-0.14107376
O	0.27991315	-0.59229440	1.81761755	C	2.44272334	1.41023170	-1.22184933
O	2.57607603	2.68590213	-1.02507204	C	3.64251424	0.53101657	-0.93750922
N	-1.31457210	0.52632380	-0.47827212	C	4.94930788	0.71346933	-1.36474037
N	1.90797942	-0.47968844	0.23273860	H	5.20204032	1.56577297	-1.98604289
C	-3.11258122	-3.37385466	-0.66345443	C	5.92456460	-0.21270251	-0.98900098
H	-2.07211591	-3.59426443	-0.43142111	H	6.94895063	-0.09296814	-1.32286375
H	-3.59831833	-4.29604330	-0.99230157	C	5.57180129	-1.29844783	-0.18548662
H	-3.61730986	-3.03105858	0.24309062	H	6.32730077	-2.02087931	0.10353786
C	-3.23444002	-2.32804882	-1.77163419	C	4.26009589	-1.48207451	0.26202374
C	-2.46069115	-2.70954699	-3.03390046	H	4.00381927	-2.31982633	0.88958404
H	-2.52875661	-1.91279095	-3.77867471	C	3.29562157	-0.54498010	-0.11708253
H	-2.90228579	-3.61286441	-3.46222995	C	1.42089085	-1.11800631	1.36963138
H	-1.41190689	-2.90532603	-2.81705977	C	-0.35945740	-1.08659858	3.07116954
C	-4.70207836	-2.05481886	-2.09894904	C	-0.74695343	-2.55956970	2.93276264
H	-5.25281301	-1.75739547	-1.20379795	H	-1.35013433	-2.71250387	2.03563977
H	-5.16174890	-2.96130539	-2.49868468	H	-1.34744561	-2.84957987	3.79906583
H	-4.79159804	-1.26371805	-2.84649145	H	0.12912622	-3.20372526	2.88580399
C	-1.50672804	-0.80087288	-0.87288235	C	-1.60734289	-0.21108315	3.17612224
C	-2.30260306	1.46786486	-0.12791397	H	-1.33707216	0.84574690	3.22073444
C	-3.69921524	1.39801829	-0.10823318	H	-2.15525626	-0.46701564	4.08578170
H	-4.22357117	0.50923353	-0.41811267	H	-2.26682871	-0.36577602	2.32077938
C	-4.40392342	2.52077935	0.32181947	C	0.57146272	-0.82990668	4.25814446
H	-5.48727824	2.47973090	0.34130783	H	1.46629633	-1.44778632	4.20806188
C	-3.75025449	3.69682009	0.72320323	H	0.03938384	-1.06186578	5.18437920
H	-4.33295469	4.55167353	1.04693334	H	0.86506848	0.22235004	4.29134825
C	-2.36288219	3.76556199	0.70611152	O	1.15057176	-0.08283815	-2.01468233
H	-1.84088255	4.66416621	1.01346503	O	1.91430924	1.04393272	-2.61006379

3f							
-1 1				C	-1.34115701	2.32326673	1.45220982
O	-1.13448533	-1.43195964	-0.88428082	C	0.05970273	1.91947140	1.23167463
O	-3.05142145	-0.27803052	-1.28192013	C	0.01821799	1.00450264	0.18304527
O	1.07781903	2.35986592	1.86570999	C	1.20505527	0.62624088	-0.60578595
O	2.40556883	-2.67114018	0.43505350	H	-0.61335768	1.36541396	-2.31942479
O	0.76065189	-1.45241311	1.39855619	C	2.24578419	1.78845486	-0.85060039
O	1.96038770	2.93726622	-1.10202110	C	3.56039482	1.15293004	-0.87452562
N	-1.34798572	0.78519949	-0.25394124	C	4.79844276	1.71135964	-1.18707912
N	2.08354615	-0.49087958	-0.17910070	H	4.86256066	2.75587614	-1.46975106
C	-3.97263177	-2.54612720	-0.91239174	C	5.92920528	0.90538065	-1.12690354
H	-3.02462605	-3.02416472	-0.67209504	H	6.90511930	1.30726929	-1.37046142
H	-4.63946883	-3.29607638	-1.34555615	C	5.79733055	-0.43784971	-0.75143228
H	-4.43042374	-2.17743287	0.00891320	H	6.68132019	-1.06447213	-0.70711033
C	-3.78995648	-1.40451914	-1.91340346	C	4.56277814	-1.00768776	-0.43574340
C	-3.06889866	-1.84773973	-3.18759580	H	4.48872540	-2.04600908	-0.15541564
H	-2.91113826	-0.99489227	-3.85260278	C	3.42893917	-0.19249968	-0.49718921
H	-3.68986254	-2.57745103	-3.71358169	C	1.75786666	-1.64778191	0.55245648
H	-2.10725088	-2.30624353	-2.96365390	C	0.25859738	-2.54162497	2.28743684
C	-5.13653906	-0.76467291	-2.25284826	C	-0.18885596	-3.75396581	1.47024913
H	-5.64677454	-0.42670467	-1.34816999	H	-0.86989228	-3.44180428	0.67761028
H	-5.77482506	-1.49600051	-2.75354118	H	-0.71762228	-4.44501264	2.13210619
H	-5.00162755	0.08967894	-2.91986128	H	0.65694995	-4.27774187	1.02808195
C	-1.80237415	-0.42424904	-0.80408868	C	-0.93758241	-1.87796372	2.96816761
C	-2.16762665	1.64086421	0.53784294	H	-0.62790927	-0.97679328	3.50143045
C	-3.54733455	1.85548200	0.52727161	H	-1.38026214	-2.57094712	3.68717223
H	-4.19123275	1.34264405	-0.16890421	H	-1.69769313	-1.60658919	2.23364187
C	-4.07742700	2.77182021	1.43779329	C	1.34161574	-2.88733000	3.31044477
H	-5.14636106	2.95511789	1.43207886	H	2.20874307	-3.34336752	2.83428885
C	-3.26098921	3.46226283	2.34457874	H	0.93372660	-3.59436741	4.03751696
H	-3.70345051	4.17169707	3.03522937	H	1.65968421	-1.99148903	3.84945141
C	-1.88719837	3.23754149	2.35466771	O	0.86313442	0.19478144	-1.97924413
H	-1.23860692	3.76066112	3.04915423	O	0.26265444	1.27357559	-2.73494864

**1**			
0 1			
O	-1.33424543	1.10663366	1.51646973
O	-3.29284117	1.05098183	0.36910686
O	1.06660148	-2.20681276	-1.54972769
O	3.99435730	-1.08695170	0.10398133
O	1.97442054	-1.40552932	1.09352188
O	-0.33732322	2.70144699	-0.69667138
N	-1.51303588	-0.03937450	-0.47838292
N	2.22406963	0.28734113	-0.38341603
C	-3.55190667	3.21613849	1.53624217
H	-2.56858231	3.20611890	2.00328504
H	-4.23075351	3.79490080	2.16740474
H	-3.48210240	3.71418191	0.56658076
C	-4.11432363	1.80514439	1.37516019
C	-4.15772084	1.02700182	2.68915836
H	-4.49749215	0.00274787	2.51829277
H	-4.86966676	1.51208035	3.36145021
H	-3.18449842	1.00413464	3.17723418
C	-5.48862055	1.83698808	0.71021633
H	-5.43760781	2.33186641	-0.26189605
H	-6.18530616	2.39074445	1.34299971
H	-5.88051922	0.82697510	0.57265173
C	-2.01567631	0.76674313	0.58001749
C	-2.24340169	-1.06933935	-1.14916558
C	-3.61216687	-1.20880954	-1.35970335
H	-4.31703399	-0.47519173	-1.00510340
C	-4.04769595	-2.33593609	-2.06078588
H	-5.11058863	-2.45586769	-2.23675983
C	-3.15939208	-3.30433983	-2.54582866
H	-3.53879238	-4.16047465	-3.08989432
C	-1.79404146	-3.15637251	-2.33258391
H	-1.08140060	-3.88740591	-2.69590730
C	-1.34810527	-2.03432499	-1.63528943
C	0.01643012	-1.64759027	-1.26971434
C	-0.14013202	-0.32331514	-0.58546083
C	0.85137289	0.59139613	-0.40583147
C	0.70690261	2.07413029	-0.60685295
C	2.07751208	2.55311624	-0.80415716
C	2.54218415	3.84104431	-1.06856844
H	1.84114889	4.66104102	-1.17075398
C	3.91260787	4.03721612	-1.19435132
H	4.30602742	5.02358018	-1.40722440
C	4.78920332	2.95334655	-1.05044475
H	5.85576584	3.11751079	-1.15348334
C	4.33640063	1.65915881	-0.78285743
H	5.02614981	0.83655772	-0.67914347
C	2.96194240	1.47454324	-0.66195170
C	2.82729888	-0.81513600	0.27403527
C	2.35575782	-2.59546419	1.92288789
C	2.81764434	-3.74247972	1.02585453
H	2.06791635	-3.95813395	0.26291458
H	2.94998072	-4.63462536	1.64315910
H	3.76487270	-3.51689149	0.53846816
C	1.03813259	-2.93688395	2.61490680
H	0.68481117	-2.09440072	3.21313711
H	1.18538024	-3.79321257	3.27648385
H	0.27057539	-3.19458403	1.88227829
C	3.41663960	-2.17178956	2.93758642
H	4.35642927	-1.91419658	2.45061999
H	3.59996931	-3.00050128	3.62592732
H	3.06924934	-1.31598899	3.52131598

Reference

[1]Gruene, T., Hahn, H. W., Luebben, A. V., Meilleur, F. & Sheldrick, G. M. 2014, J. Appl. Cryst. 47, 462-466. DOI: 10.1107/S2053229614024218
